# Deconstructing Synaptotagmin-1's Distinct Roles in Synaptic Vesicle Priming and Neurotransmitter Release

**DOI:** 10.1523/JNEUROSCI.1945-21.2022

**Published:** 2022-04-06

**Authors:** Boris Bouazza-Arostegui, Marcial Camacho, Marisa M. Brockmann, Sina Zobel, Christian Rosenmund

**Affiliations:** ^1^Institute of Neurophysiology, Charité–Universitätsmedizin Berlin, corporate member of Freie Universität Berlin and Humboldt-Universität zu Berlin, 10117 Berlin, Germany; ^2^NeuroCure Cluster of Excellence, Charité–Universitätsmedizin Berlin, 10117 Berlin, Germany

**Keywords:** autaptic culture, development, synaptic transmission, synaptic vesicle priming, Synaptotagmin

## Abstract

Synaptotagmin-1 (SYT1) is a synaptic vesicle resident protein that interacts via its C2 domain with anionic lipids from the plasma membrane in a calcium-dependent manner to efficiently trigger rapid neurotransmitter (NT) release. In addition, SYT1 acts as a negative regulator of spontaneous NT release and regulates synaptic vesicle (SV) priming. How these functions relate to each other mechanistically and what role other synaptotagmin (SYT) isoforms play in supporting and complementing the role of SYT1 is still under intensive investigation. In this work, we analyzed three putative functions of SYT1 in exocytosis by systematically varying its expression in autaptic hippocampal glutamatergic neurons from mice of either sex. We find that regulation of release probability is most sensitive to variation of expression levels, whereas its impact on vesicle priming is least sensitive. Also, loss of SYT1 phenotypes on spontaneous release and vesicle priming is compensated in less mature synaptic cultures by redundant support from SYT7. Overall, our data help in resolving some controversies in SYT1 functions in exocytosis and in our understanding of how SYT1 contributes to the pathophysiology underlying SYT1-related human neurologic disorders.

**SIGNIFICANCE STATEMENT** Our work clarifies the functions of SYT1 protein in synaptic vesicle priming and spontaneous and calcium-evoked neurotransmitter release and analyzes whether these occur at different stages of synaptic responses by examining their relative sensitivity to protein concentration at the synaptic terminal. We demonstrate that these synaptic functions are unequally sensitive to both protein levels and neuronal stage, indicating that they operate under distinct molecular mechanisms. Furthermore, we analyze how these functions are modulated by another synaptotagmin isoform expression. We show that to understand the phenotype displayed by SYT1 knock-out neurons (*Syt1*^−/−^) is necessary to consider the interplay between SYT1 and SYT7 molecules at the presynaptic terminal.

## Introduction

Calcium-evoked neurotransmitter (NT) release from the presynaptic nerve terminal occurs in less than 1 ms following the activation of voltage-gated calcium channels triggered by the arrival of an action potential (AP; Katz, 1969; Sabatini and Regehr, 1999). A key element in orchestrating the rapid and efficient presynaptic Ca^2+^-evoked NT release is the synaptic vesicular protein Synaptotagmin-1 (SYT1; DiAntonio and Schwarz, 1994; Fernández-Chacón et al., 2001; Rizo and Xu, 2015). Biochemical studies have demonstrated that SYT1 is a phospholipid-binding machine that acts in a calcium-dependent manner via its C2A and C2B cytoplasmic domains to trigger NT release (Perin et al., 1990; Brose et al., 1992; Sutton et al., 1995; Chapman and Davis, 1998; Fernandez et al., 2001).

In *Drosophila* and mammalian synapses in the absence of *Syt1*, AP-evoked synchronous release is drastically impaired (Broadie et al., 1994; Geppert et al., 1994; Yoshihara and Littleton, 2002; Nishiki and Augustine, 2004; Maximov and Südhof, 2005; Xue et al., 2008), but structure/function analysis of the C2B domain reveals that SYT1 is involved in two additional functions. SYT1 also acts as a clamping factor, suppressing spontaneous release of synaptic vesicles (SVs; DiAntonio and Schwarz, 1994; Littleton et al., 1994; Chicka et al., 2008; Xu et al., 2009), and different research groups have shown a role for SYT1 in SV docking/priming, mediating the recruitment of vesicles to the plasma membrane (PM) by the interaction with phosphatidylserine and phosphatidylinositol 4, 5-bisphosphate (Reist et al., 1998; Liu et al., 2009; Bacaj et al., 2015; Chang et al., 2018). However, how these functions of SYT1 mechanistically interact is currently unclear.

In addition to the different roles of SYT1 in the process of neurotransmitter release, other synaptotagmin isoforms have been implicated in distinct and overlapping roles. For example, the SYT7 isoform has been claimed to be a high-affinity calcium sensor for asynchronous release (Bacaj et al., 2013; Chen and Jonas, 2017), a regulator of short-term plasticity (STP; Wen et al., 2010; Jackman et al., 2016; Chen et al., 2017b; Fujii et al., 2021), and to act redundantly with SYT1 in the maintenance of the ready releasable pool (RRP) of SVs (Bacaj et al., 2015). Furthermore, the expression of synaptotagmin isoforms is regulated in a cell-type-specific manner. At the calyx of Held synapse and in some GABAergic neurons, the vesicular SYT2 protein, the closest relative isoform of SYT1 (Geppert et al., 1991; Marquèze et al., 1995), regulates neurotransmission redundantly with SYT1 (Pang et al., 2006; Chen et al., 2017a). Synaptic transmission depends on SYT1 in the early postnatal calyx of Held synapses but later switches to SYT2 (Kochubey et al., 2016), suggesting that dynamic changes in synaptotagmin content at synapses during development and their redundant functions in priming and release have an impact in synaptic function. Consistent with this idea, heterozygotic *de novo* mutations in the *syt1* gene detected in patients have been shown to be associated with a neurodevelopmental disorder (Baker et al., 2015, 2018; Bradberry et al., 2020).

In this study, we therefore aim to analyze the proposed distinct roles of SYT1 in vesicle priming and spontaneous and evoked release, and investigate these functions in the context of development, its interplay with SYT7, and the relative contribution of SYT1 expression levels on these functions. We found that the loss of function phenotype of SYT1 depends on the maturation stage of neurons, where immature neurons can compensate the loss of SYT1 with expression of SYT7 in two of the three SYT1 functions. Furthermore, by systematically investigating the three key functions of SYT1 as a function of protein levels, we define distinct sensitivity of these processes, arguing a distinct role of SYT1 in these different functions.

## Materials and Methods

### Animals

In this study, we used embryonic day (E)18–19 *Syt1*^+/+^, *Syt1*^+/−^, and *Syt1*^−/−^ embryos on C57BL/6 background of either sex generated by interbreeding *Syt1* heterozygous mice as described previously (Xue et al., 2008). All procedures and animals used were handled according to the regulations of Directive 2010/63/EU of the European Parliament on the protection of animals used for scientific purposes and were approved by the Berlin state authorities under the license number G-Project 106/20 and the animal welfare committee of Charité–Universitätsmedizin Berlin.

### Lentiviral constructs and production

Lentiviruses were generated by the Viral Core Facility of the Charité–Universitätsmedizin Berlin. All lentiviral particles were produced as described previously (Lois et al., 2002). Lentiviral *Syt1* construct used for rescue and overexpression experiments was generated from mouse *Syt1* cDNA (National Center for Biotechnology Information reference sequence NM_001252341). The cDNA was cloned into a lentiviral vector (FUGW) with a human synapsin1 promoter and following a nuclear localization signal (NLS)-RFP-P2A expression cassette for identification of infected cells. A lentivirus expressing only NLS-RFP-P2A controlled by the human synapsin1 promoter served as control. For short hairpin RNA (shRNA)-mediated knockdown experiments of SYT1 protein, an shRNA cassette containing a 19 bp target sequence of *Syt1* (5′AGTCTTCCTGCTGCCCGAC-3′) was cloned downstream of a U6 promoter that also contained a ubiquitin promoter-driven RFP expression cassette (f(U6)shRNA-Syt1.Ubi-RFP.WPRE). For shRNA mediated knockdown of SYT7 a 21 bp targeting sequence KD607 from Bacaj et al. (2013) was used under the control of U6 promoter expression cassette within a lentivirus that also contained a human synapsin1 promoter to control the nuclear RFP expression cassette (f(U6) shRNA-Syt7.Syn-NLS-RFP.WPRE). A scramble RNA (*scRNA*) sequence served in both shRNA constructs as control (Watanabe et al., 2014). The lentiviral titer for all generated constructs was estimated by quantification of hippocampal neurons in mass culture expressing a fluorescent marker after day *in vitro* (DIV)7.

### Autaptic neuronal culture and viral infection

For electrophysiology recordings and immunocytochemistry experiments, autaptic glutamatergic hippocampal neurons were prepared as previously reported by Bekkers and Stevens (1991). Briefly, hippocampal neurons derived from *Syt1*^+/+^, *Syt1*^+/−^, and *Syt1*^−/−^ embryonic mice (E18–19) of either sex were used and plated at a density of 300 cells/cm^2^ on 30 mm coverslips containing astrocytic microislands. Astrocytes were obtained from cerebral cortices of postnatal day (P)0–2 C57BL/6N mice and plated at a density of 5000 cells/cm^2^ on the micropattern coverslips 1 week before of the preparation of the neurons. Autaptic neuronal cultures were infected with the appropriate viral construct 24–48 h after plating and maintained at 37°C and 5% CO_2_.

### Electrophysiology

Whole-cell patch-clamp recordings in autaptic neurons were performed at DIV11–21. All electrophysiological recordings were done at room temperature with a MultiClamp 700B amplifier (Molecular Devices) controlled by Clampex 10 software (Molecular Devices). Data were digitally sampled at 10 kHz and were filtered using a low-pass Bessel filter at 3 kHz. Series resistance was compensated up to 70%. Autaptic cultures during recordings were immersed in an extracellular solution that contained the following (in mm): 140 NaCl, 2.4 KCl, 10 HEPES, 10 glucose, 2 CaCl_2_ and 4 MgCl_2_. Borosilicate glass patch pipettes were pulled using a multistep puller (model P-1000, Sutter Instruments). Pipettes with resistance (3–5 MOhms) were filled with KCl-based intracellular solution containing the following (in mm): 136 KCl, 17.8 HEPES, 1 EGTA, 4.6 MgCl_2_, 4 ATP-Na_2_, 0.3 GTP-Na_2_, 12 creatine phosphate, and 50 U/ml phosphocreatine kinase. Both internal and extracellular solutions were adjusted to 300 mOsm, pH 7.4. Neurons were clamped at −70 mV during recordings. Exclusion criteria were stablished for patched cells with a leak current higher than −200 pA. Single APs were evoked by a 2 ms depolarization step to 0 mV and EPSCs were recorded. To measure the synchronicity of synaptic responses we inverted the EPSC charge and integrated the signal. Then, we fitted a two**-**components function provided by AxoGraph X (AxoGraph Scientific), from which we calculated their relative contribution to the total charge released. The first component represents the fast and synchronous release part of the EPSC charge and the second component the slow and asynchronous release part of the EPSC charge.

The RRP of synaptic vesicles was assessed by the application for 5 s of external solution containing 500 mm sucrose (Rosenmund and Stevens, 1996). Evoked-sucrose currents were recorded, and the RRP size was estimated by integrating the area of the evoked-sucrose current with the steady-state current set as the baseline. Vesicular release probability (Pvr) was calculated as the ratio between the charge of the EPSC and the evoked-sucrose charge.

We calculated the paired-pulsed ratio (PPR) by dividing the second EPSC (EPSC2) amplitude by the first (EPSC1). A train of AP stimulation at 20 Hz for 5 s was recorded to assess short**-**term plasticity. The synaptic responses from the train of AP were normalized to the first EPSC peak amplitude.

To analyze mini EPSCs (mEPSCs), electrophysiological traces were filtered at 1 kHz, and the range of parameters for inclusion of selected events using a conventionally defined template algorithm in AxoGraph X (AxoGraph Scientific) were 5–200 pA, 0.15–1.5 ms rise time, and 0.5–5 ms half-width. False-positive events were excluded by subtracting events detected from traces in the presence of AMPA receptor antagonist, NBQX (3 μm). Spontaneous release rate was calculated by dividing the mEPSC frequency by the number of synaptic vesicles in the RRP. The number of synaptic vesicles in the RRP was calculated by dividing the RRP size by the mEPSC charge. Off-line analysis was performed using AxoGraph X (AxoGraph Scientific).

### Immunocytochemistry and image acquisition

Autaptic hippocampal neurons were immunostained as previously reported by Xue et al. (2007). Briefly, neurons were fixed with 4% paraformaldehyde (PFA; Sigma-Aldrich) for 10 min at DIV11 and DIV16 (see Fig. 3; see Figs. 2, 5, 6, 7 and 8 for 15–21 DIV). Primary antibodies monoclonal mouse anti-Synaptotagmin-1 (1:1000; Synaptic Systems), polyclonal guinea pig anti-VGLUT1 (1:4000; Synaptic Systems), and polyclonal rabbit anti-Synaptogamin 7 (1:500; Synaptic Systems) were used. Secondary antibodies (1:500) conjugated with Alexa Fluor 405, 488, or 647 (Jackson ImmunoResearch) were used to visualize fluorescence. For quantitative assessment, all groups compared in one experiment were processed in parallel using identical antibodies solutions and other reagents.

Single neurons on the astrocytic micro islands were imaged by using a Nikon Scanning Confocal A1Rsi+ with a 60×, 1.4 NA oil-immersion objective. For acquiring all the images, we used the same microscope and camera with identical acquisition settings for all experimental groups. Overexposure and photobleaching were avoided by checking the fluorescence signal saturation in synaptic boutons. Z stacks of neurons were set with a 0.3 μm interstack interval and total *z* axis range of 5–6 μm, and a sum of intensity projection was further used for analyses. The images were obtained at 1024 × 1024 pixels resolution and at the pixel size of 0.2 μm. During acquisition, laser settings were applied identically to all coverslips quantified in all experimental groups compared.

The analysis was performed using ImageJ software (National Institutes of Health) by drawing regions of interst (ROIs) of 50 synapses per neuron. Excitatory synapses (ROIs) were defined by staining for the SV marker VGLUT1. SYT1 or SYT7 fluorescence intensity signal for each synapse was measured within the defined ROI. Five to 10 autaptic neurons were collected per condition per culture, and at least three independent cultures were analyzed per experiment. Relative expression level of SYT1 among groups was calculated by normalizing the measured intensities of SYT1 to that of VGLUT1. The data were normalized to the control of each experiment.

### Experimental design and statistics

For electrophysiology and immunocytochemistry experiments, we recorded and imaged approximately the same number of autaptic neurons from each experimental group each day to reduce data variability. For each parameter analyzed, the number of neurons used (*n*) and the number of independent cultures (*N*) are indicated in the figures, specifically within the bar graphs (*n*/*N*).

Data were acquired from at least three independent hippocampal autaptic cultures generated from three different animals (*N* = 3) to minimize culture–culture variation. We chose the nonlinear regression model standard Hill equation (see Fig. 7). We did not constrain any parameter of the model to a constant value. We performed a global nonlinear regression; we specify that parameters are shared to fit all datasets. All data points are weighted equally in the model.

Data from each experimental group were pooled except for those that were normalized to the mean value of the control group (see Figs. 2, 7, 8). Statistical analyses were performed using Prism 8 (GraphPad). All data were first subjected to Pearson omnibus K2 normality testing. Two-tailed unpaired *t* test or one-way ANOVA test for normally distributed data and Mann–Whitney test or Kruskal–Wallis ANOVA test for non-normally distributed data were then conducted. *Post hoc* multiple-comparison methods were used following ANOVA tests. Significance and *p* values were calculated and are shown in the corresponding figures or Table 1.

**Table 1.** Summary table with the electrophysiological parameters measured in autaptic hippocampal glutamatergic neurons related to the culture stage

		SYT1^−/−^			SYT1^−/−^ +SYT7KD				
		DIV			DIV		SYT1 expression (DIV15–21)		
		11–12	15–16	20–21	11–12	15–16	<50%	50–100%	>100%
Synaptic transmission parameter	RRP	=	Reduced	Reduced	Reduced	Reduced	Reduced	=	=
	Spontaneous release	=	Increased	Increased	Increased	Increased	Increased	Increased	=
	Evoked release	Reduced	Reduced	Reduced	Reduced	Reduced	Reduced	=	=
	Pvr	Reduced	Reduced	Reduced	=	=	Reduced	Reduced	*Increased

*The Pvr endency toward an increased value, confirmed by STP experiment. The “=” symbol is “not significant differences detected when compared to control group”.

## Results

### Phenotype of synaptotagmin-1 loss in synaptic vesicle priming and spontaneous release is sensitive to neuronal maturation state

Given the controversy over SYT1 involvement in clamping and priming SVs, the observation of developmentally dependent effects of SYT2 in the calyx of Held (Kochubey et al., 2016), and the discovery of new point mutations in *SYT1* gene associated with a neurodevelopmental disorder (Baker et al., 2018), we aimed to reanalyze the roles of Synaptotagmin-1 in SV priming, synchronized and spontaneous release as a function of neuronal culture age. We recorded from autaptic hippocampal neurons derived from *Syt1*^+/+^ or *Syt1*^−/−^ mice across a 10 d time period (DIV11–21), and subsequently grouped our results into three different classes (DIV11–12, 15–16 and 20–21).

**Figure 1.**
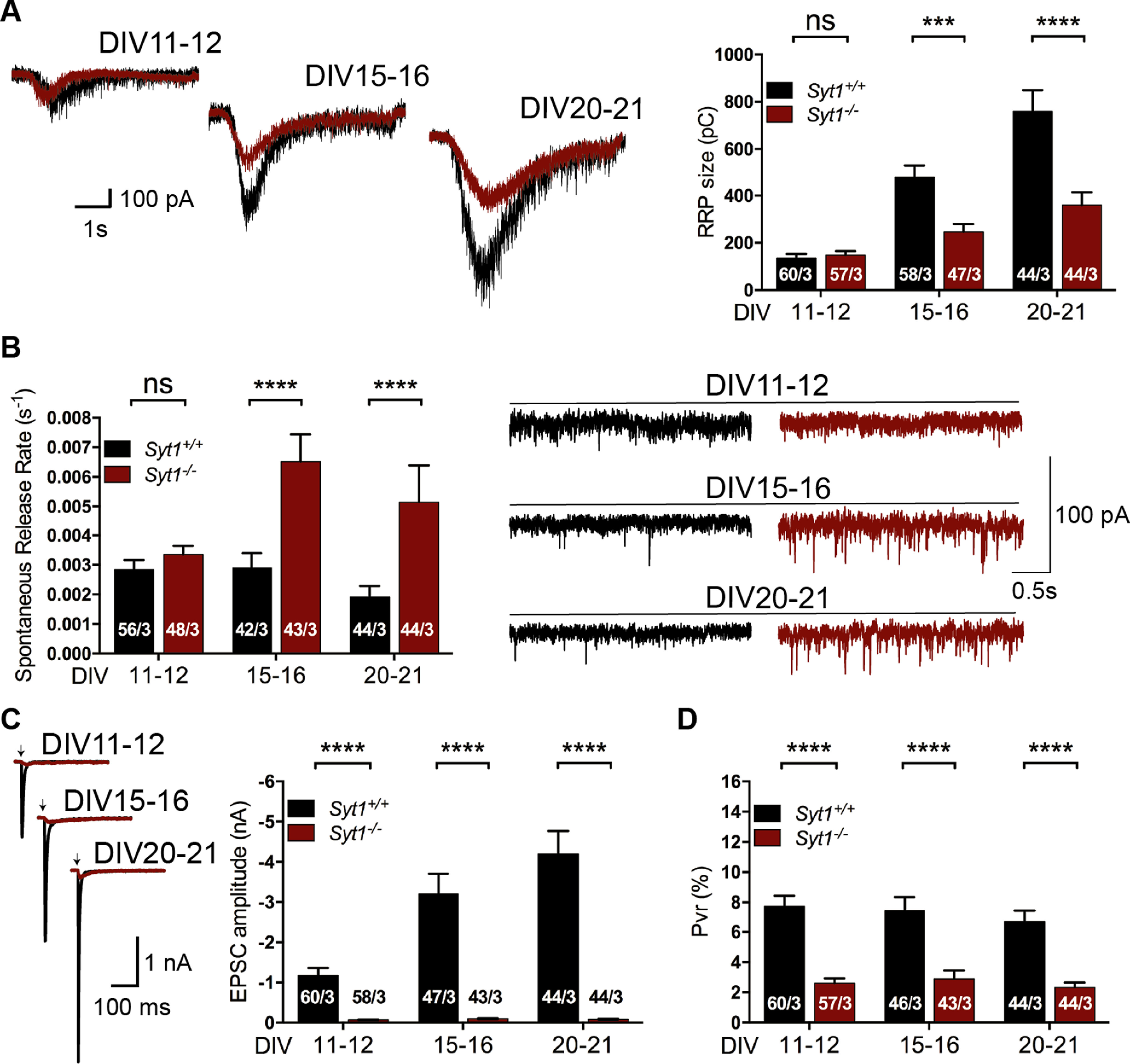
Electrophysiological characterization of *Synaptotagmin-1* knock-out hippocampal glutamatergic autaptic neurons at different time points. ***A***, Representative sucrose-evoked current traces (left) and summary bar graphs (right) of total current charge of *Syt1*^+/+^ (black) and *Syt1*^−/−^ (red) neurons evoked by 0.5 m sucrose solution for 5 s at early (DIV11–12), intermediate (DIV15–16), and late (DIV20–21) culture stages. ***B***, Example traces of spontaneous release events (right) and summary bar graphs of spontaneous release rate (left) calculated by dividing the mEPSC frequency by the number of primed synaptic vesicles at different neuronal culture stages. ***C***, Representative EPSC traces (left) and summary bar graphs of EPSC amplitudes (right) evoked by a 2 ms depolarization in 2 mm extracellular Ca^2+^ from autaptic neurons at the three neuronal stages from *Syt1*^+/+^ (black) and *Syt1*^−/−^ (red). Action potentials were blanked for better EPSC illustration and substituted by arrows. ***D***, Plot of Pvr in percentage calculated by dividing the evoked EPSC charge by the sucrose charge at the different neuronal stages. Data are mean ± SEM. Statistical significance and *p* values were calculated using the Mann–Whitney *U* test (***p ≤ 0.001, ****p ≤ 0.0001). ns, Not significant.

To assess SV priming, we measured the RRP of SVs using 5 s application of 500 mOsm hypertonic solution (Rosenmund and Stevens, 1996) and integrated the transient component of the evoked inward current. Although the RRP charge of *Syt1*^+/+^ and *Syt1*^−/−^ autaptic neurons at DIV11–12 was indistinguishable, at both later culturing stages (DIV15–16) and (DIV20–21) we detected a significant (∼40–50%) reduction in RRP charge in the *Syt1*^−/−^ group (Fig. 1*A*; RRP, pC: DIV11–12, *Syt1*^+/+^ 135 ± 18, *n* = 60/3, and *Syt1*^−/−^ 148 ± 17, *n* = 57/3, *p* = 0.26; DIV15–16, *Syt1*^+/+^ 477 ± 54, *n* = 58/3, and *Syt1*^−/−^ 247 ± 33, *n* = 47/3, *p* = 0.0004; DIV20–21, *Syt1*^+/+^ 759 ± 89, *n* = 44/3, and *Syt1*^−/−^ 360 ± 54, *n* = 44/3, *p* < 0.0001; Mann–Whitney test). Next, we analyzed spontaneous neurotransmitter release. We observed that loss of SYT1 led to no significant changes in spontaneous release rates for the early DIV group, whereas spontaneous release rate was enhanced in the two older DIV groups (Fig. 1*B*; Spontaneous rate, *s*^−1^: DIV11–12, *Syt1*^+/+^ 0.0028 ± 0.0003, *n* = 56/3, and *Syt1*^−/−^ 0.0034 ± 0.0003, *n* = 48/3, *p* = 0.0544; DIV15–16, *Syt1*^+/+^ 0.0029 ± 0.0005, *n* = 42/3, and *Syt1*^−/−^ 0.0065 ± 0.0009, *n* = 43/3, *p* < 0.0001; DIV20-21, *Syt1*^+/+^ 0.0019 ± 0.0004, *n* = 44/3, and *Syt1*^−/−^ 0.0052±0.0012, *n* = 44/3, *p* < 0.0001; Mann–Whitney test), emphasizing that the impact of SYT1 loss on spontaneous release, like the impact on RRP size, is dependent on the maturation state of the neuron.

In contrast, Ca^2+^-evoked release was massively affected regardless of culture age as indicated by the reduction in EPSC amplitude and charge in all three DIV groups (Fig. 1*C*; EPSC amplitude, nA: DIV11–12, *Syt1*^+/+^ −1.17 ± 0.19, *n* = 60/3, and *Syt1*^−/−^ −0.069 ± 0.007, *n* = 58/3, *p* < 0.0001; DIV15–16, *Syt1*^+/+^ −3.2 ± 0.5, *n* = 47/3, and *Syt1*^−/−^ −0.097 ± 0.012, *n* = 43/3, *p* < 0.0001; DIV20–21, *Syt1*^+/+^ −4.19 ± 0.58, *n* = 44/3, and *Syt1*^−/−^ −0.085 ± 0.012, *n* = 44/3, *p* < 0.0001; Mann–Whitney test). Moreover, the Pvr, as calculated by dividing the EPSC charge by the RRP charge, was similarly reduced by ∼60% in all three DIV groups measured (Fig. 1D; Pvr, %: DIV11–12, *Syt1*^+/+^ 7.7 ± 0.7, *n* = 60/3, and *Syt1*^−/−^ 2.6 ± 0.3, *n* = 57/3, *p* < 0.0001; DIV15–16, *Syt1*^+/+^ 7.4 ± 0.9, *n* = 46/3, and *Syt1*^−/−^ 2.9 ± 0.6, *n* = 43/3, *p* < 0.0001; DIV20–21, *Syt1*^+/+^ 6.7 ± 0.7, *n* = 44/3, and *Syt1*^−/−^ 2.3 ± 0.3, *n* = 44/3, *p* < 0.0001; Mann–Whitney test).

**Figure 2.**
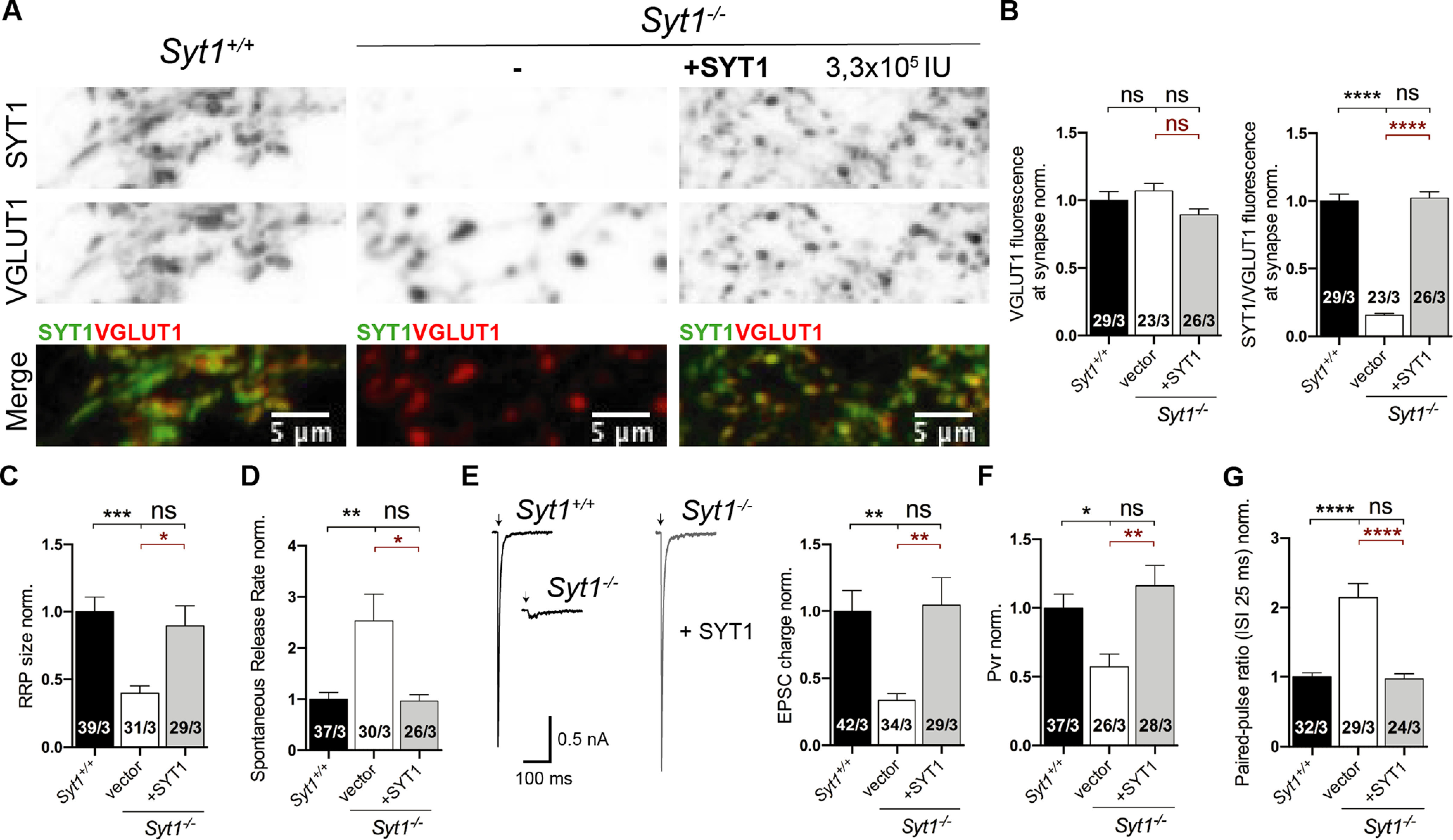
Evaluation of the synaptic SYT1 functions in *Syt1*^−/−^ neurons rescued with SYT1. All immunocytochemistry and electrophysiological experiments were done from *Syt1*^+/+^ and *Syt1*^−/−^ hippocampal glutamatergic autaptic neurons at DIV15–21. ***A***, Representative images of hippocampal glutamatergic autaptic neurons of *Syt1*^−/−^ infected with a lentivirus containing a nuclear GFP vector (−) or *Syt1*. ***B***, Right, Summary bar graph showing SYT1/VGLUT1 fluorescence normalized to *Syt1*^+/+^. Summary bar plot of VGLUT1 fluorescence (left) and normalized summary bar plot of SYT1/VGLUT1 fluorescence intensity. ***C***, Bar plot of RRP size estimated by application of a hypertonic solution of *Syt1*^−/−^ neurons rescued with SYT1 normalized to the *Syt1*^+/+^control. ***D***, Summary bar plot of spontaneous release rate from *Syt1*^−/−^ neurons rescued with SYT1 normalized to the *Syt1*^+/+^control. ***E***, Sample traces of EPSCs (right) and summary bar plot of the quantification of total EPSC charge transfer (left) from *Syt1*^−/−^ neurons rescued with SYT1 normalized to the *Syt1*^+/+^control. Artifacts and/or action potentials were blanked and substituted with arrows. ***F***, Bar plot of Pvr from *Syt1*^−/−^ neurons rescued with SYT1 normalized to *Syt1*^+/+^ control. ***G***, Graph of the paired-pulse ratio. All data shown indicate mean ± SEM. Statistical analysis was applied by Kruskal–Wallis test (*p < 0.05, **p < 0.01, ***p < 0.001, ****p <0.0001. ns, Not significant. Scale bars: 5 μm.

Lentiviral-mediated SYT1 rescue experiments showed fully restored SV priming and spontaneous and AP-induced release efficacy in stages DIV15–21 (Fig. 2; RRP normalized (norm.): *Syt1*^+/+^ 1 ± 0.11, *n* = 39/3, *Syt1*^−/−^ 0.40 ± 0.05, *n* = 31/3, *p* < 0.001, and *Syt1*^−/−^_+*SYT1*_ 0.93 ± 0.15, *n* = 29/3, *p* = n.s. or <0.05; Spontaneous rate norm.: *Syt1*^+/+^ 1 ± 0.13, *n* = 37/3, *Syt1*^−/−^ 2.53 ± 0.53, *n* = 30/3, *p* < 0.01, and *Syt1*^−/−^_+*SYT1*_ 0.96 ± 0.12, *n* = 26/3, *p* = n.s. or <0.05; EPSC charge norm.: *Syt1*^+/+^ 1 ± 0.16, *n* = 42/3, *Syt1*^−/−^ 0.34 ± 0.05, *n* = 34/3, *p* < 0.01, and *Syt1*^−/−^_+*SYT1*_ 1.05 ± 0.2, *n* = 29/3, *p* = n.s. or <0.01; Pvr norm.: *Syt1*^+/+^ 1 ± 0.10, *n* = 37/3, *Syt1*^−/−^ 0.57 ± 0.09, *n* = 26/3, *p* < 0.05, and *Syt1*^−/−^_+*SYT1*_ 1.16 ± 0.15, *n* = 28/3, *p* = n.s. or <0.01; PPR 40 Hz, norm.: *Syt1*^+/+^ 1 ± 0.06, *n* = 32/3, *Syt1*^−/−^ 2.15 ± 0.20, *n* = 29/3, *p* < 0.0001, and *Syt1*^−/−^_+*SYT1*_ 0.97 ± 0.07, *n* = 24/3, *p* = n.s. or <0.0001; Kruskal–Wallis test), indicating that the loss of function phenotype of SYT1 at older cultures was because of the loss of the protein and not because of developmental processes. Expression levels of exogenously expressed SYT1 were near wild-type levels as indicated by relative SYT1 immunofluorescence intensity from VGLUT1 marker positive compartments (Fig. 2*A*,*B*; VGLUT1 norm.: *Syt1*^+/+^ 1 ± 0.06, *n* = 29/3, *Syt1*^−/−^ 1.07 ± 0.06, *n* = 23/3, *p* = n.s., and *Syt1*^−/−^_+*SYT1*_ 0.89 ± 0.04, *n* = 26/3, *p* = n.s./n.s.; SYT1/VGLUT1 norm.: *Syt1*^+/+^ 1 ± 0.05, *n* = 29/3, *Syt1*^−/−^ 0.16 ± 0.01, *n* = 23/3, *p* < 0.0001, and *Syt1*^−/−^_+*SYT1*_ 1.02 ± 0.05, *n* = 26/3, *p* = n.s. or <0.0001; Kruskal–Wallis test).

Overall, our data confirm that loss of SYT1 leads to loss of synchronized and efficient AP-triggered release but also that the impairment of RRP and clamping of spontaneous release only appears in more mature neurons. One possible explanation could be that the loss of SYT1 at early maturation stages may be compensated by other synaptotagmin isoform, which would be downregulated over neuronal development as similarly shown at the calyx of Held synapses (Kochubey et al., 2016). These findings may provide an explanation for findings in autaptic glutamatergic neurons where no significant changes in RRP size were reported when recordings were pooled over extended times *in vitro* (e.g., Liu et al., 2009; DIV12–17). Therefore, in the remaining part of the experiments, we limited our experiments to neurons that were DIV15 or older.

### Partial functional redundancy between synaptotagmin-1 and synaptotagmin-7

**Figure 3.**
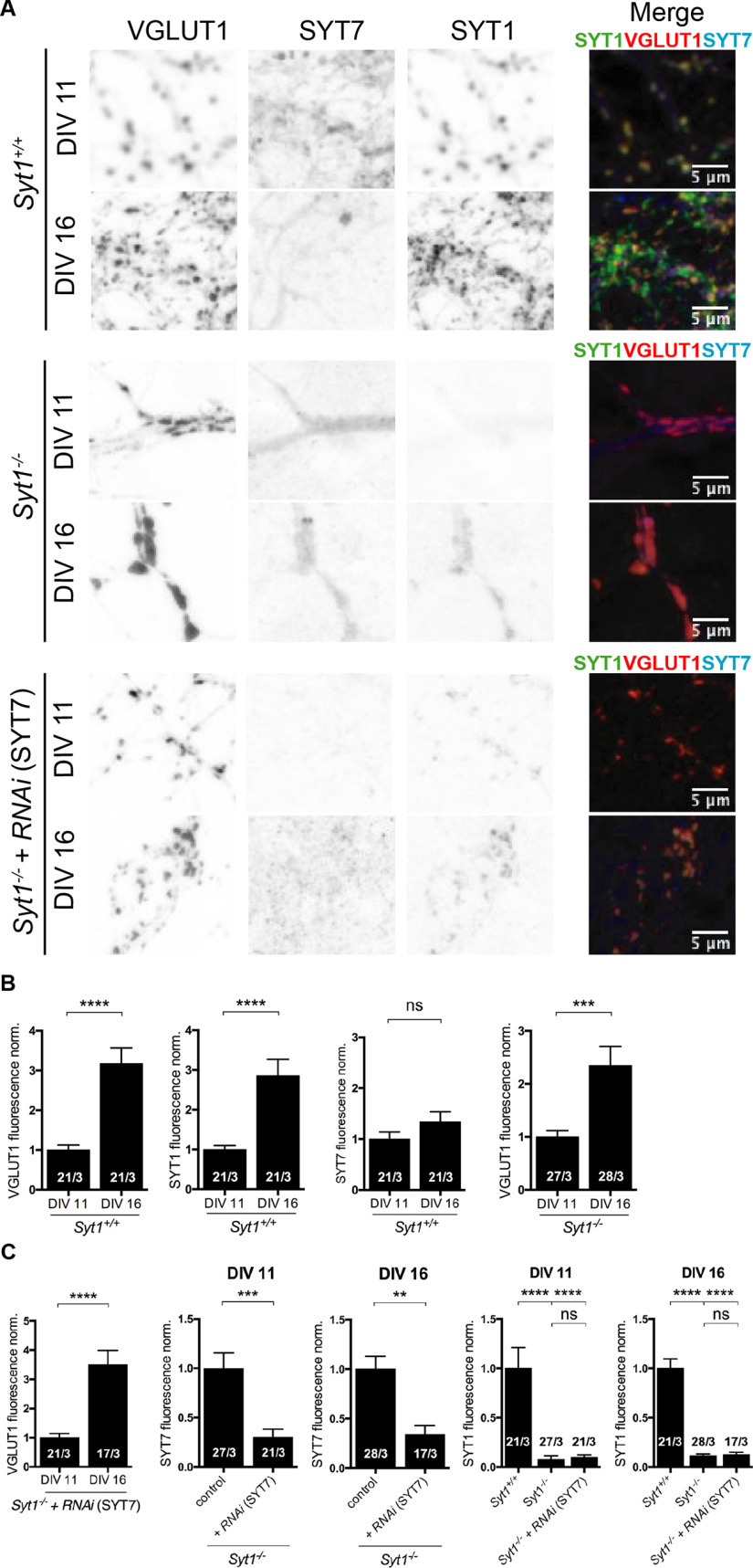
Presynaptic SYT7 quantification of Synaptotagmin-7 in presence and absence of SYT1 and its silencing effect in *Syt1*^−/−^ hippocampal glutamatergic neurons at different neuronal stages. ***A***, ***B***, Representative images (***A***) and **(*B*)** quantification of SYT1 (green) and SYT7 (blue) protein levels at VGLUT1 (red) positive regions at DIV11 and DIV16 of *Syt1*^+/+^ and *Syt1*^−/−^ neurons. ***C***, Quantification of SYT1, SYT7, and VLGUT1 from *Syt1*^−/−^ autaptic neurons infected with lentivirus carrying either an empty vector or RNA interference (*RNAi*) for SYT7 at early and mature stages. Data are mean ± SEM. Statistical significance and *p* values were calculated using the Mann–Whitney *U* test (**p ≤ 0.01,***p ≤ 0.001, ****p ≤ 0.0001). ns, Not significant. Scale bars: 5 μm.

Recent genetic and functional analysis indicates that SYT7 plays a redundant role in SV priming function (Bacaj et al., 2015). Moreover, the expression of the Synaptotagmin1/2 paralogs are developmentally regulated at a murine auditory synapse, contributing to maturation-dependent variation in synaptic phenotypes in synaptotagmin loss of function mouse models (Kochubey et al., 2016). We therefore explored whether SYT7 expression in less mature hippocampal neurons contributes to the milder phenotype of SYT1 loss by knocking down SYT7 protein expression in *Syt1*^−/−^ neurons. First, we examined SYT1 and SYT7 protein levels in presynaptic compartments at early (DIV11–12) or intermediate stages (DIV15–16) in *Syt1*^+/+^ and *Syt1*^−/−^ neurons and subsequently measured at both stages the presynaptic SYT7 reduction affected by lentiviral expression of a SYT7 *shRNA* construct in *Syt1*^−/−^ autaptic neurons (Fig. 3). We used VGLUT1 protein expression to spatially define presynaptic compartments and to serve as a reference signal for SYT1 and SYT7 expression levels. In *Syt1*^+/+^ neurons, SYT1 protein levels gradually increased with time of DIV but stayed relatively constant in comparison to VGLUT1. In contrast, SYT7 protein expression levels remained constant and, consequently, SYT7/VGLUT1 signal decreased over time in culture (Fig. 3*A*,*B*; VGLUT1 norm.: *Syt1*^+/+^*_DIV11_* 1 ± 0.12, *n* = 21/3, and *Syt1*^+/+^*_DIV16_* 3.2 ± 0.4, *n* = 21/3, *p* < 0.0001; two-tailed unpaired *t* test; SYT1 norm.: *Syt1*^+/+^*_DIV11_* 1 ± 0.10, *n* = 21/3, and *Syt1*^+/+^*_DIV16_* 2.9 ± 0.4, *n* = 21/3, *p* < 0.0001; two-tailed unpaired *t* test; SYT7 norm.: *Syt1*^+/+^*_DIV11_* 1 ± 0.14, *n* = 21/3, and *Syt1*^+/+^*_DIV16_* 1.3 ± 0.2, *n* = 21/3, *p* = n.s.; Mann–Whitney test; VGLUT1 norm.: *Syt1*^−/−^*_DIV11_* 1 ± 0.12, *n* = 27/3, and *Syt1*^+/+^*_DIV16_* 2.3 ± 0.36, *n* = 28/3, *p* < 0.001; Mann–Whitney test). We tested how effective the SYT7 *shRNA* reduced the presynaptic SYT7 protein expression and found an average reduction of ∼75% across all DIVs tested using *Syt1*^−/−^ autaptic neurons (Fig. 3*C*; VGLUT1 norm.: *Syt1*^−/−^_+*RNAi(Syt7)DIV11*_ 1 ± 0.14, *n* = 21/3, and *Syt1*^−/−^_+*RNAi(Syt7)DIV16*_ 3.5 ± 0.47, *n* = 17/3, *p* < 0.0001; Mann–Whitney test; SYT7DIV11 norm.: *Syt1*^−/−^ 1 ± 0.21, *n* = 27/3, and *Syt1*^−/−^_+*RNAi(Syt7)*_ 0.30 ± 0.15, *n* = 21/3, *p* < 0.001; Mann–Whitney test; SYT7DIV16 norm.: *Syt1*^−/−^ 1 ± 0.13, *n* = 28/3, and *Syt1*^−/−^_+*RNAi(Syt7)*_ 0.34 ± 0.09, *n* = 17/3, *p* < 0.001; Mann–Whitney test; SYT1DIV11 norm.: *Syt1*^+/+^ 1 ± 0.20, *n* = 21/3, *Syt1*^−/−^ 0.08 ± 0.04, *n* = 27/3, *p* < 0.0001, and *Syt1*^−/−^_+*RNAi(Syt7)*_ 0.1 ± 0.096, *n* = 21/3, *p* < 0.0001; Kruskal–Wallis test; SYT1DIV16 norm.: *Syt1*^+/+^ 1 ± 0.096, *n* = 21/3, *Syt1*^−/−^ 0.11 ± 0.02, *n* = 28/3, *p* < 0.0001, and *Syt1*^−/−^_+*RNAi(Syt7)*_ 0.07 ± 0.06, *n* = 17/3, *p* < 0.0001; Kruskal–Wallis test).

**Figure 4.**
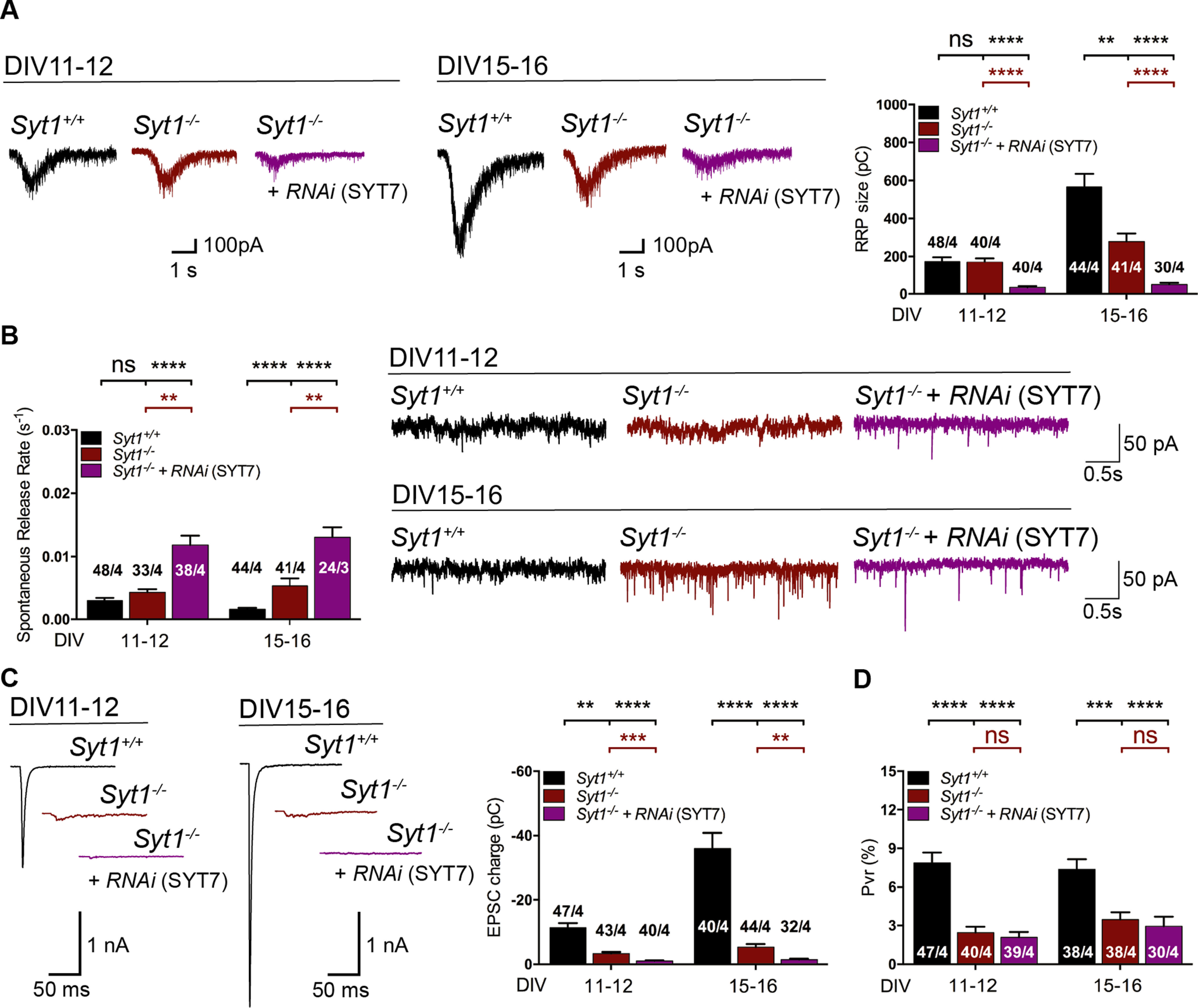
Knockdown of Synaptotagmin-7 in *Syt*1^−/−^ hippocampal glutamatergic neurons at different neuronal stages. ***A***, Representative traces at early (DIV11–12) and intermediate (DIV15–16) autaptic neuronal stages (right) and summary bar graphs (left) of the charge of sucrose-evoked release of *Syt1*^+/+^ (black), *Syt1*^−/−^ (red), and *Syt1*^−/−^ neurons infected with lentivirus containing RNAi for silencing SYT7 (purple). ***B***, Example traces of spontaneous release events (right) and summary bar graphs of spontaneous release rate (left) at the two different neuronal stages as ***A***. ***C***, Representative EPSC traces (right) and summary bar graphs of total EPSC charge measured over an interval of 1 s (left). ***D***, Summary bar graphs of vesicular release probability. Data indicate mean ± SEM. Statistical significance and *p* values were estimated by a Kruskal–Wallis test (*p ≤ 0.05, **p ≤ 0.01, ***p ≤ 0.001, ****p ≤ 0.0001). ns, Not significant.

Reduction of SYT7 protein expression in *Syt1*^−/−^ autaptic neurons reduced RRP size and increased mEPSC rates at DIV11–12 and DIV15–16 (Fig. 4*A*,*B*; RRP, pC: DIV11–12, *Syt1*^+/+^ 165 ± 23, *n* = 48/4, *Syt1*^−/−^ 170 ± 21, *n* = 40/4, *p* = n.s., and *Syt1*^−/−^_+*iRNA(Syt7)*_ 34 ± 6, *n* = 40/4, *p* < 0.0001/0.0001; DIV15–16, *Syt1*^+/+^ 567 ± 69, *n* = 44/4, *Syt1*^−/−^ 279 ± 43, *n* = 41/4, *p* < 0.01, and *Syt1*^−/−^_+*iRNA(Syt7)*_ 50 ± 9, *n* = 30/4, *p* < 0.0001/0.0001; Kruskal–Wallis test; Spontaneous rate, *s*^−1^: DIV11–12, *Syt1*^+/+^ 0.003 ± 0.0004, *n* = 48/4, *Syt1*^−/−^ 0.0043 ± 0.0005, *n* = 33/4, *p* = n.s., and *Syt1*^−/−^_+*iRNA(Syt7)*_ 0.012 ± 0.0015, *n* = 38/4, *p* < 0.0001/0.01; DIV15–16, *Syt1*^+/+^ 0.0016 ± 0.0002, *n* = 44/4, *Syt1*^−/−^ 0.0054 ± 0.0012, *n* = 41/4, *p* < 0.01, and *Syt1*^−/−^_+*iRNA(Syt7)*_ 0.013 ± 0.001, *n* = 24/4, *p* < 0.0001/0.01; Kruskal–Wallis test). These results suggest that SYT7 compensates for the loss of SYT1-dependent synaptic vesicle priming and clamp of spontaneous release (Fig. 4*A*,*B*). Next, we examined whether SYT7 expression contributes evoked NT release and the probability of vesicle fusion of *Syt1*^−/−^ neurons. Although knocking down SYT7 expression levels in *Syt1*^−/−^ neurons had an impact on the amplitude and charge of the Ca^2+^-evoked release at both stages, the changes were proportional to those observed for the RRP size and thus did not lead to a further significant effect in the Pvr (Fig. 4*C*,*D*; EPSC charge, pC: DIV11–12, *Syt1*^+/+^ −11.1 ± 1.5, *n* = *47/4, Syt1*^−/−^ −3.8 ± 0.5, *n* = 43/4, *p* < 0.01, and *Syt1*^−/−^_+*iRNA(Syt7)*_ −1.1 ± 0.2, *n* = 40/4, *p* < 0.0001/0.001; DIV15–16, *Syt1*^+/+^ −36 ± 5, *n* = 40/4, *Syt1*^−/−^ −5.3 ± 1, *n* = 44/4, *p* < 0.0001, and *Syt1*^−/−^_+*iRNA(Syt7)*_ −1.4 ± 0.3, *n* = 32/4, *p* < 0.0001/0.01; Pvr, %, DIV11–12: *Syt1*^+/+^ 8.5 ± 1, *n* = 47/4, *Syt1*^−/−^ 3.2 ± 0.5, *n* = 40/4, *p* < 0.0001, and *Syt1*^−/−^_+*iRNA(Syt7)*_ 3.3± 0.6, *n* = 39/4, *p* < 0.0001/n.s.; DIV15–16, *Syt1*^+/+^ 7.4 ± 0.8, *n* = 38/4, *Syt1*^−/−^ 3.5 ± 0.6, *n* = 38/4, *p* < 0.001, and *Syt1*^−/−^_+*iRNA (Syt7)*_ 2.9 ± 0.8, *n* = 30/4, *p* < 0.0001/n.s.; Kruskal–Wallis test) of *Syt1*^−/−^ autaptic neurons in any stages tested.

Altogether these results suggest that although SYT1 is essential for evoked neurotransmitter release, SYT7 is well capable of substituting in the role of SYT1 in clamping spontaneous release and enabling SV priming, which results in the weakening of SYT1 deficiency of these two phenotypes in less mature neurons.

### Synaptotagmin-1 haploinsufficiency affects release efficiency and spontaneous release rate

Our results on SV priming and spontaneous clamping suggest that the underlying phenotypes are dependent on both SYT1 and SYT7 protein expression levels. Because monoallelic mutations in *SYT1* lead to neurologic disorders (Baker et al., 2015, 2018), it raises the question of whether and which of the three SYT1 functions in NT release is impaired by reduced SYT1 protein amount. We first quantified SV priming, clamping, and calcium-evoked release in *Syt1*^+/+^, *Syt1*^+/−^, and *Syt1*^−/−^ at a maturation stage where SYT1 dominates the synaptic phenotype (DIV15–21).

**Figure 5.**
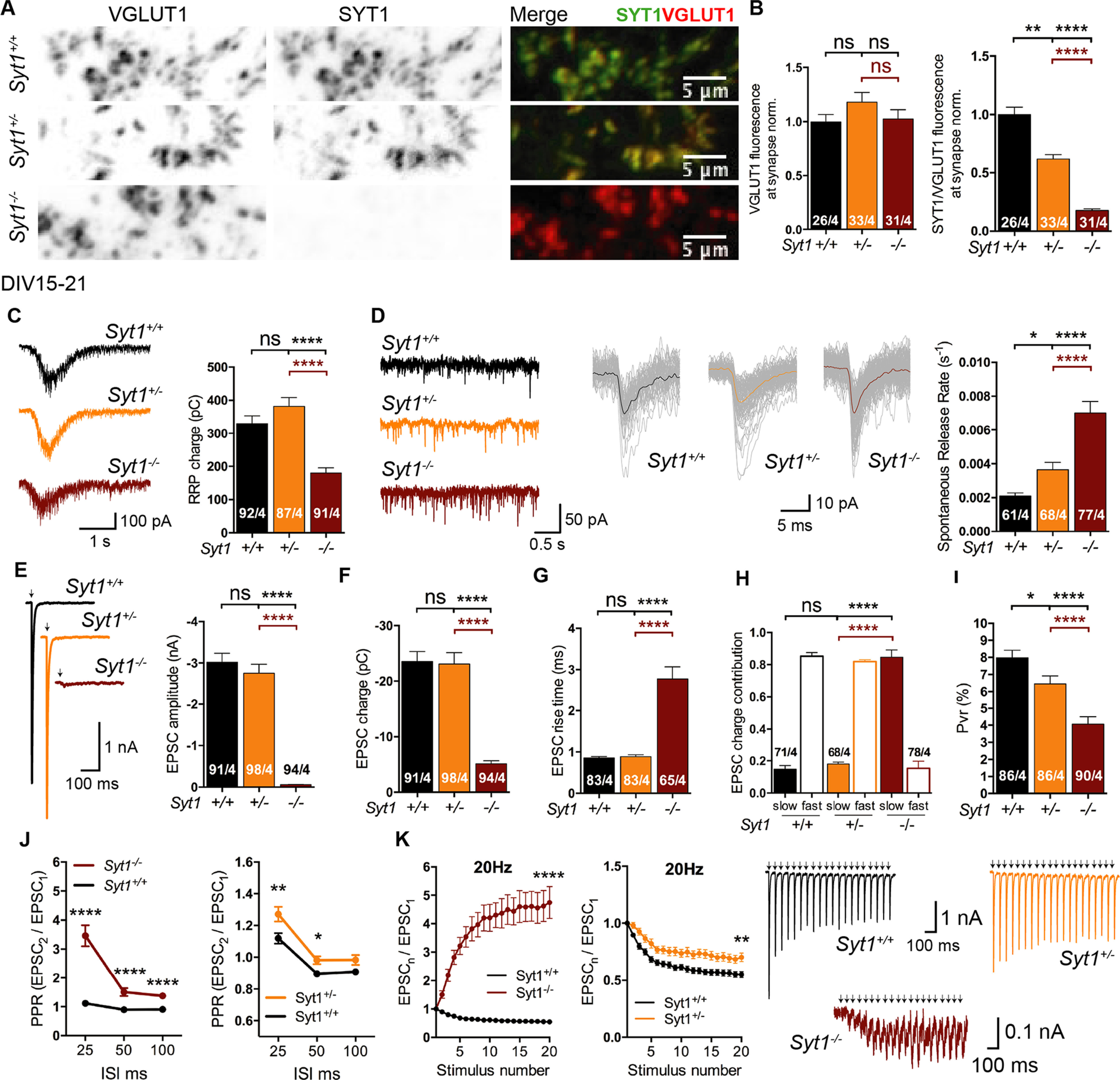
Evaluation of SV priming and neurotransmitter release in Synaptotagmin-1 heterozygous neurons. All immunocytochemistry and electrophysiological experiments were done from *Syt1*^+/+^, *Syt*1^+/−^, and *Syt1*^−/−^ hippocampal glutamatergic autaptic neurons at DIV15-21. ***A***, Sample images from *Syt1*^+/+^, *Syt1*^+/−^ and *Syt1*^−/−^ hippocampal glutamatergic autaptic neurons double labeled with VGLUT1 (left, black puncta), SYT1 (middle, black puncta), antibodies and colocalization image of SYT1 in green and VLGUT1 in red (right, Merge), demonstrating the presence of Synaptotagmin-1 at the presynaptic terminals. Scale bar, 5 µm. ***B***, Bar plot of VGLUT1 average fluorescence intensities at presynaptic terminal for the three *Syt-1* genotypes (left), normalized to SYT1 expression levels of the *Syt1*^+/+^ neurons. Summary bar plot of SYT1 average fluorescence intensity at VGLUT1-positive puncta normalized to SYT1 average fluorescence intensity of *Syt1*^+/+^ (right). ***C***, Left, Sample traces of sucrose responses for the three *Syt-1* genotypes. Right, Bar graph of sucrose-evoked current charges of *Syt1*^+/+^ (black), *Syt1*^+/−^ (orange), and *Syt1*^−/−^ (cayenne) autaptic neurons. ***D***, Left, Example traces of spontaneous release events and 106 mEPSC events superimposed from *Syt1*^+/+^, 198 mEPSC events superimposed from *Syt1*^+/−^, and 257 mEPSC events superimposed from *Syt1*^−/−^ of a recording time period of 3 s in a 15 ms time window (center). Right, Summary bar graph of the spontaneous release rate of mEPSC events. ***E***, Representative EPSC traces (left) and summary bar graphs (right) of EPSC amplitudes. ***F***, Summary bar graph of total EPSC charge transfer. ***G***, Summary bar plot of the rise time (20–80%) of EPSCs. ***H***, Bar graphs of the relative contribution of the synchronic and asynchronic components to the EPSC charge. ***I***, Bar plot of the average vesicular release probability of *Syt1*^+/+^, *Syt*1^+/−^ and *Syt1*^−/−^. ***J***, Summary graph of the average paired-pulse ratio plotted against interpulse interval from 25 to 100 ms corresponding to 40, 20, and 10 Hz of *Syt1*^+/+^ (black), *Syt1*^+/−^ (orange), and *Syt1*^−/−^ (cayenne) autaptic neurons. ***K***, Short-term plasticity response during high-frequency (20 Hz) stimulation. Representative sample traces of EPSCs resulted from the stimulation of 20 consecutives APs separated by 50 ms (right) and plots of mean EPSC amplitudes normalized to the first EPSC of *Syt1*^+/+^ (black), *Syt1*^+/−^ (orange), and *Syt1*^−/−^ (cayenne) during train stimulation. Artifacts and action potentials were blanked from the traces and substituted by arrows in ***E*** and ***K***. Data are mean ± SEM. Statistical significances and *p* values were obtained by Kruskal–Wallis test (*p ≤ 0.05, **p ≤ 0.01, ***p ≤ 0.001, ****p ≤ 0.0001). ns, Not significant. Scale bars: 5 μm.

Immunocytochemistry experiments showed that the amount of SYT1 protein at presynaptic terminals of *Syt1*^+/−^ neurons was ∼50% reduced compared with *Syt1*^+/+^ neurons (Fig. 5*A*,*B*; *VGLUT1* norm.: *Syt1*^+/+^ 1 ± 0.07, *n* = 26/4, *Syt1*^+/−^ 1.18 ± 0.09, *n* = 33/4, *p* = n.s., and *Syt1*^−/−^ 1.03 ± 0.09, *n* = 31/4, *p* = n.s./n.s.; *SYT1/VGLUT1* norm.: *Syt1*^+/+^ 1 ± 0.06, *n* = 26/4, *Syt1*^+/−^ 0.62 ± 0.04, *n* = 33/4, *p* < 0.01, and *Syt1*^−/−^ 0.18 ± 0.02, *n* = 31/4, *p* < 0.0001/< 0.0001; Kruskal–Wallis test). As expected, the complete absence of SYT1 in *Syt1*^−/−^ neurons resulted in a ∼40% reduction in the pool size (Fig. 5*C*). However, we found no significant differences in the RRP size between *Syt1*^+/+^ and *Syt1*^+/−^ neurons (Fig. 5*C*; RRP, pC: *Syt1*^+/+^ 330 ± 24, *n* = 92/4, *Syt1*^+/−^ 382 ± 26, *n* = 87/4, *p* = n.s., and *Syt1*^−/−^ 180 ± 15, *n* = 91/4, *p* < 0.0001/<0.0001; Kruskal–Wallis test). This indicates that loss of >50% of SYT1 protein is required to impair the role of SYT1 in SV priming. A significant increase in the spontaneous release rate of synaptic vesicles was observed in *Syt1*^+/−^ autaptic neurons (Fig. 5*D*; Spontaneous rate, *s*^−1^: *Syt1*^+/+^ 0.002 ± 0.0002, *n* = 61/4, *Syt1*^+/−^ 0.004 ± 0.0004, *n* = 68/4, *p* < 0.05, and *Syt1*^−/−^ 0.007 ± 0.0007, *n* = 77/4, *p* < 0.0001/< 0.0001; Kruskal–Wallis test).

In agreement with previous work (Geppert et al., 1994; Nishiki and Augustine, 2004; Xue et al., 2008), autaptic *Syt1*^−/−^ neurons showed severe desynchronization of evoked release, which is reflected in the drastic reduction in peak EPSC amplitude (Fig. 5*E*). Furthermore, integrating the charge of the EPSC over 1 s past AP triggering, we also observed an ∼75% decrease in the EPSC charge (Fig. 5*F*). In contrast, we detected no significant difference in *Syt1*^+/−^ EPSC amplitude and charge when compared with *Syt1*^+/+^ autaptic neurons (Fig. 5*E*,*F*; EPSC amplitude, nA: *Syt1*^+/+^ −3.0 ± 0.2, *n* = 91/4, *Syt1*^+/−^ −2.8 ± 0.2, *n* = 98/4, *p* = n.s., and *Syt1*^−/−^ −0.06 ± 0.006, *n* = 94/4, *p* < 0.0001/<0.0001; Kruskal–Wallis test; EPSC charge, pC: *Syt1*^+/+^ −24 ± 2, *n* = 91/4, *Syt1*^+/−^ −23 ± 2, *n* = 98/4, *p* = n.s., and *Syt1*^−/−^ −5 ± 0.5, *n* = 94/4, *p* < 0.0001/<0.0001; Kruskal–Wallis test). We then examined whether 50% loss of SYT1 protein leads to some changes in EPSC kinetics in the *Syt1*^+/−^ neurons. The EPSC rise time of *Syt1*^+/−^ neurons was like wild type, whereas the rise time was about threefold slower for *Syt1*^−/−^ neurons (Fig. 5*G*; EPSC rise time, ms: *Syt1*^+/+^ 0.9 ± 0.04, *n* = 83/4, *Syt1*^+/−^ 0.9 ± 0.04, *n* = 83/4, *p* = n.s., and *Syt1*^−/−^ *2.8*±*0.3*, n = 65/4, *p* < 0.0001/< 0.0001; Kruskal–Wallis test). The decay time of EPSC was analyzed by a two-exponential fit, and we found that between the *Syt1*^+/+^ and *Syt1*^+/−^ neurons, both the fast and slow components were unaltered in their relative amplitudes and time constants (Fig. 5*H*). In contrast, in *Syt1*^−/−^ neurons, time constant increased, and the fractional contribution of the slow component increased from ∼20% to 80% (Fig. 5*H*; EPSC components: *Syt1*^+/+^, slow 0.15 ± 0.02, fast 0.85 ± 0.02, *n* = 71/4, *Syt1*^+/−^, slow 0.18 ± 0.01, fast 0.82 ± 0.01, *n* = 68/4, *p* = 0.1705, and *Syt1*^−/−^, slow 0.85 ± 0.05, fast 0.15 ± 0.05, *n* = 78/4, *p* < 0.0001/< 0.0001; Kruskal–Wallis test).

The reduction of EPSC charge in *Syt1*^−/−^ exceeds the reduction in RRP size, indicating that complete loss of SYT1 leads to a reduction in vesicular release probability. We thus measured vesicular release probability in *Syt1*^+/−^ neurons and surprisingly found a significant reduction in Pvr when compared with *Syt1*^+/+^ autaptic neurons (Fig. 5*I*; Pvr, %: *Syt1*^+/+^ 8.6 ± 0.4, *n* = 86/4, *Syt1*^+/−^ 7.5 ± 0.6, *n* = 86/4, *p* < 0.05, and *Syt1*^−/−^ 4.4 ± 0.5, *n* = 90/4, *p* <0.0001/< 0.0001; Kruskal–Wallis test). The reduced release probability was corroborated by analysis of short-term plasticity experiments, in which we applied sequential pairs of action potentials with an interpulse interval of 25, 50, or 100 ms. Consistent with the Pvr results, *Syt1*^−/−^ neurons displayed a significant increase of the PPR at 10, 20, and 40 Hz compared with *Syt1*^+/+^ and a significant increase of the PPR at 20 and 40 Hz compared with *Syt1*^+/−^ (Fig. 5*J*; 10 Hz: *Syt1*^+/+^ 0.91 ± 0.02, *n* = 47/4, *Syt1*^+/−^ 0.98 ± 0.03, *n* = 62/4, *p* = n.s., and *Syt1*^−/−^ 1.38 ± 0.09, *n* = 41/4, *p* < 0.0001/< 0.0001; Kruskal–Wallis test; 20 Hz: *Syt1*^+/+^ 0.90 ± 0.02, *n* = 53/4, *Syt1*^+/−^ 0.99 ± 0.02, *n* = 50/4, *p* < 0.05, and *Syt1*^−/−^ 1.51 ± 0.13, *n* = 20/4, *p* < 0.0001/< 0.0001; Kruskal–Wallis test; 40 Hz: *Syt1*^+/+^ 1.09 ± 0.03, *n* = 86/4, *Syt1*^+/−^ 1.28 ± 0.05, *n* = 81/4, *p* < 0.01, and *Syt1*^−/−^ 3.46 ± 0.37, *n* = 78/4, *p* < 0.0001/< 0.0001; Kruskal–Wallis test). In addition, we performed a 20 AP at 20 Hz train pulses experiment. *Syt1*^−/−^ neurons strongly facilitated, and even *Syt1*^+/−^ neurons showed significantly less depression compared with *Syt1*^+/+^ neurons (Fig. 5*K*; *Syt1*^+/+^ 0.55 ± 0.03, *n* = 53/4, *Syt1*^+/−^ 0.70 ± 0.04, *n* = 50/4, *p* < 0.01, and *Syt1*^−/−^ 4.7 ± 0.6, *n* = 20/4, *p* < 0.0001; Kruskal–Wallis test).

Together, these results demonstrate that the 50% reduced SYT1 protein at *Syt1*^+/−^ presynaptic terminals did not affect the RRP size but modestly impaired release efficiency and the rate of spontaneous release. Although it isn't clear whether the patients carrying a heterozygous mutation in the *SYT1* gene may have an altered SYT1 presence at the glutamatergic synapses, these findings may be relevant to understand how a 50% deficiency in SYT1 expression could contribute to develop an aberrant neurologic phenotype.

### Synaptotagmin-1 is a limiting factor for vesicle fusion release efficiency during high-frequency stimulation

**Figure 6.**
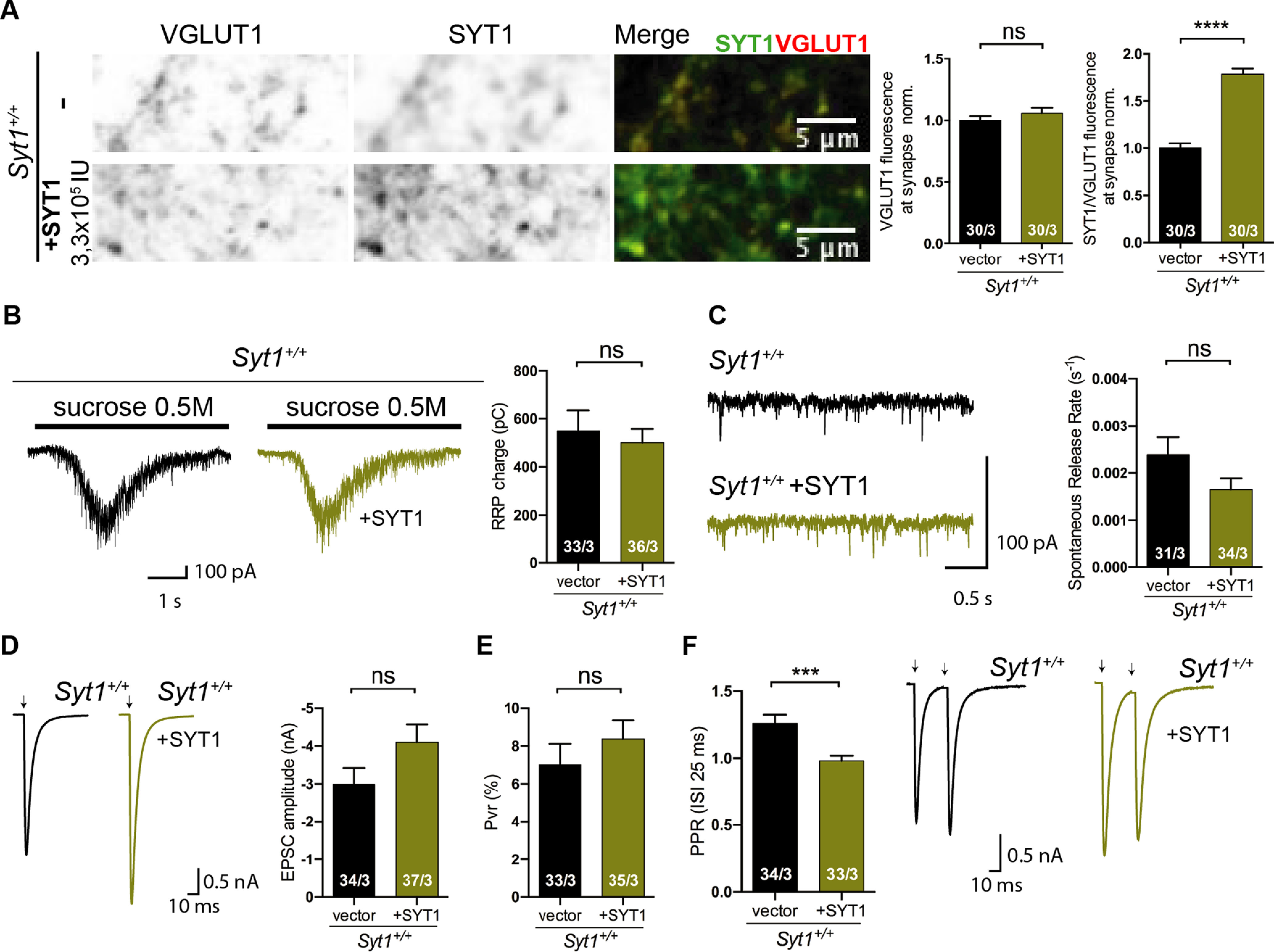
Impact of Synaptotagmin-1 overexpression on synaptic properties. All immunocytochemistry and electrophysiological experiments were done from *Syt1*^+/+^ hippocampal glutamatergic autaptic neurons at DIV15–21. ***A***, Representative images of *Syt1*^+/+^ autaptic neurons with and without overexpression of SYT1, co-immunolabeled for VGLUT1 (left), SYT1 (middle), and Merge image (right), showing the presence of VGLUT1 in red and SYT1 in green. Scale bar, 5 µm. Quantification graphs of normalized VGLUT1 fluorescence intensities and SYT1/VGLUT1 fluorescence intensity ratios of *Syt1*^+/+^ hippocampal neurons overexpressing GFP vector (black) or SYT1 (right, olive green). ***B***, Representative sucrose-evoked current traces (left) and summary bar plot of RRP size (right). ***C***, Example traces of spontaneous release events (left) and summary bar graph (right) of spontaneous release rate. ***D***, Sample EPSC traces (left) and bar plot of EPSC amplitude (right). ***E***, Summary bar graph of average probability of vesicle release. ***F***, Left, Bar graph of PPR at 40 Hz. Right, Representative sample traces of EPSCs resulted from the stimulation of two consecutives APs separated by 25 ms. Artifacts and/or action potentials are blanked and substituted with arrows in ***D*** and ***F***. All data shown are mean ± SEM. Statistical analysis was applied Mann–Whitney *U test* (*p ≤ 0.05, **p ≤ 0.01, ***p ≤ 0.001, ****p ≤ 0.0001). ns, Not significant. Scale bars, 5 μm.

Our analysis with the *Syt1*^+/−^ neurons showed that 50% reduced STY1 expression affects synaptic properties such as spontaneous release rate and efficiency of the release, indicating the relative sensitivity of SYT1 protein levels on release efficacy. We extended this analysis to overexpression of the SYT1. We used a dose of lentivirus to mediate SYT1 overexpression in *Syt1*^+/+^ autaptic neurons (DIV15–21), which led to an overexpression of ∼75% of WT levels in SYT1 content in presynaptic terminals (Fig. 6*A*; *VGLUT1* norm.: *Syt1*^+/+^ 1 ± 0.04, *n* = 30/3, and *Syt1*^+/+^_+*SYT1*_ 1.06 ± 0.04, *n* = 30/3, *p* = 0.29; *SYT1/VGLUT1* norm.: *Syt1*^+/+^ 1 ± 0.05, *n* = 30/3, and *Syt1*^+/+^_+*SYT1*_ 1.78 ± 0.06, *n* = 30/3, p < 0.0001; two-tailed unpaired *t* test). RRP size (Fig. 6*B*; RRP, pC: *Syt1*^+/+^ 549 ± 85, *n* = 33/3, and *Syt1*^+/+^_+*SYT1*_ 500 ± 57, *n* = 36/3, *p* = 0.67; Mann–Whitney test), spontaneous release rate (Fig. 6*C*; Spontaneous rate, *s*^−1^: *Syt1*^+/+^ 0.003 ± 0.0005, *n* = 31/3, and *Syt1*^+/+^_+*SYT*1_ 0.0017 ± 0.0002, *n* = 34/3, *p* = 0.45; Mann–Whitney test), and the EPSC amplitude (Fig. 6*D*; EPSC amplitude, nA: *Syt1*^+/+^ −2.99 ± 0.43, *n* = 34/3, and *Syt1*^+/+^_+*SYT1*_ −4.10 ± 0.47, *n* = 37/3, *p* = 0.084; Mann–Whitney test) were statistically not significant compared with *Syt1*^+/+^ neurons (DIV15–21). Also computing Pvr in the SYT1 overexpressing neurons did not show any significant change (Fig. 6*E*; Pvr, %: *Syt1*^+/+^ 7.0 ± 1.1, *n* = 33/3, and *Syt1*^+/+^_+*SYT1*_ 8.4 ± 1.0, *n* = 35/3, *p* = 0.19; Mann–Whitney test). However, SYT1 overexpression caused more depression of the second response during paired-pulse experiments (Fig. 6*F*; 40 Hz: *Syt1*^+/+^ 1.26 ± 0.06, *n* = 34/3, and *Syt1*^+/+^_+*SYT1*_ 0.98 ± 0.04, *n* = 33/3, *p* = 0.0005; Mann–Whitney test) compared with *Syt1*^+/+^, emphasizing that release efficacy is more sensitive to changes in SYT1 levels than its function in regulating RRP size.

### Manipulation of endogenous expression levels of synaptotagmin-1 reveals different functional thresholds

**Figure 7.**
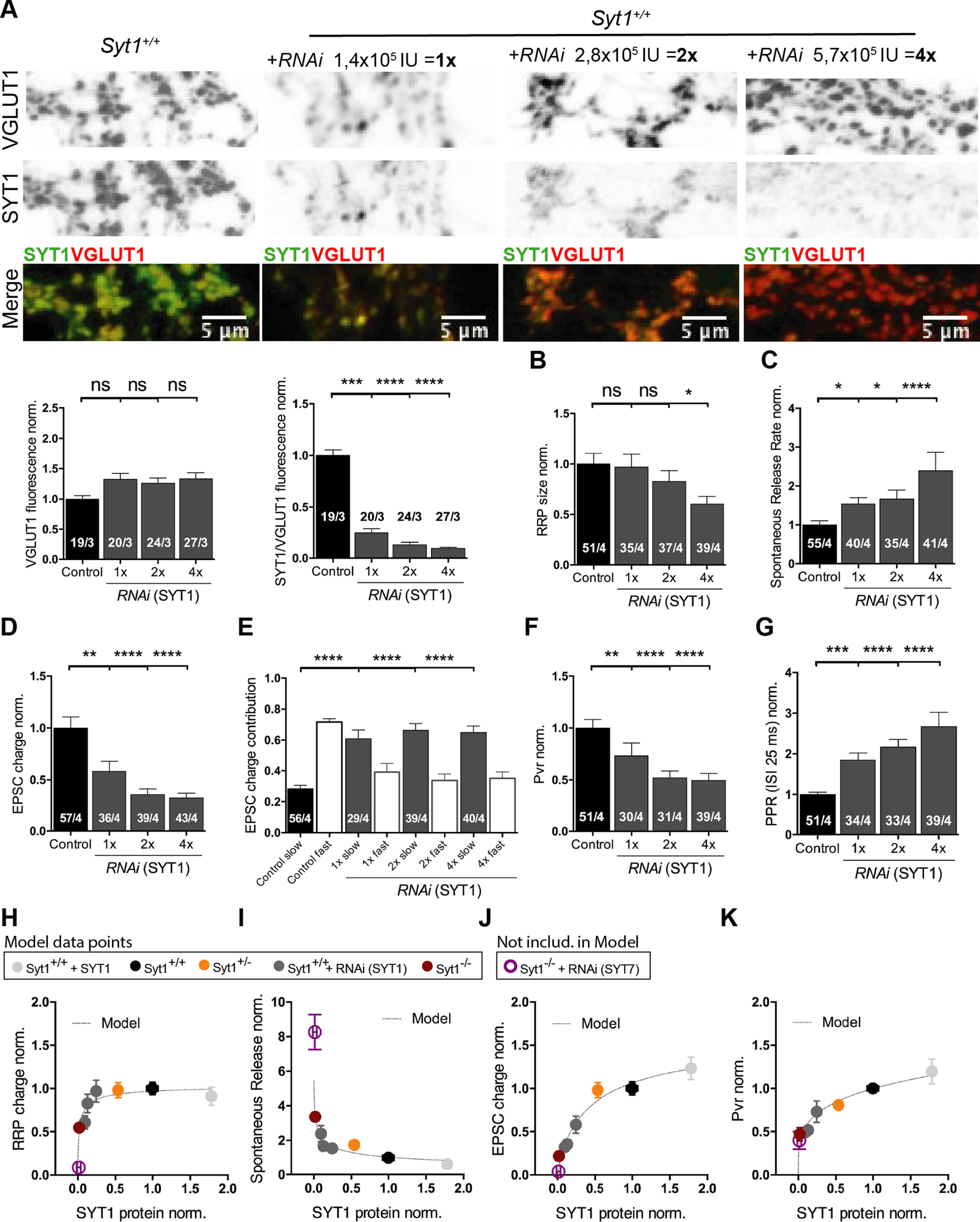
Dose dependence of Synaptotagmin-1 on neurotransmitter release functions. All immunocytochemistry and electrophysiological experiments were done from *Syt1*^+/+^ hippocampal glutamatergic autaptic neurons at DIV15–21. ***A***, Representative images immunolabeled for VGLUT and SYT1, as in Figure 6, from *Syt1*^+/+^ hippocampal glutamatergic autaptic neurons infected with a lentiviral vector expressing a scramble RNA (control) or increasing amounts of a *Syt1 RNAi*. Scale bar, 5 µm. Summary bar graphs of VGLUT1 fluorescence intensities at presynaptic terminal (left) and SYT1/VGLUT1 fluorescence intensities ratios normalized to *Syt1*^+/+^ (right). ***B***, Plot of sucrose charges of Syt1^+/+^ control and increasingly knock-down SYT1 autaptic neurons normalized to the average Syt1^+/+^ control sucrose charge. ***C***, Summary bar graph of spontaneous release rate normalized to control *Syt1*^+/+^. ***D***, Normalized bar graphs of the effect on evoked EPSC charge of titration of SYT1. ***E***, Bar graphs of the relative contribution of the synchronic and asynchronic components to the EPSC total charge transfer with different amount of SYT1 expression levels. ***F***, Vesicular release probability (Pvr) normalized to *Syt1*^+/+^. ***G***, Normalized summary graph of the paired-pulse stimulation at 40 Hz. Statistical analysis was applied by Kruskal–Wallis test (*p ≤ 0.05, **p ≤ 0.01, ***p ≤ 0.001, ****p ≤ 0.0001). ns, Not significant. ***H–K***, Plots of RRP size (***H***), spontaneous release rate (***I***), EPSC charge (***J***), and Pvr (***K***) normalized to *Syt1*^+/+^ against the SYT1/VGLUT1 fluorescence intensity ratios, obtained from the titration of SYT1 in autaptic neuronal cultures. To calculate SYT1 expression at the following synapses: *Syt1*^+/+^ (black), *Syt1*^+/−^ (orange), and *Syt1*^−/−^ (burgundy), data (Fig. 5) and *Syt1*^−/−^+ *RNAi* (SYT7, purple) data (Fig. 4, DIV16) were normalized to *Syt1*^+/+^, subtracting the *Syt1*^−/−^ expression levels in the different experiments. For *Syt1*^+/+^ with the different amounts of *Syt1 RNAi* (dark gray) and *Syt1*^+/+^ plus SYT1 (light gray) data (Figs. 6, 7) SYT1 expression levels were normalized to *Syt1*^+/+^. All cells used for the model were from DIV15–21. *Syt1*^−/−^ + *RNAi* (SYT7) cells were excluded from the model, but the group is represented in the plot to illustrate its functional relevance. The discontinuous gray line represent the curve fitting of the Hill function. All data shown represent mean ± SEM. Scale bars, 5 μm. For ***H–K*** data point values, see the table in Extended Data Figure 7-1.

Does SYT1 regulate NT release and recruitment of SVs to the presynaptic PM in a protein concentration-dependent manner? To address this question, we examined the impact of titrating endogenous SYT1 protein expression in autaptic neurons at DIV15–21 using RNA interference (RNAi) technology, which led to a graded reduction of SYT1 levels in presynaptic terminals down to 10–25% of *Syt1*^+/+^ SYT1 expression levels (Fig. 7*A*; *VGLUT1* norm.: *Syt1*^+/+^ 1 ± 0.06, *n* = 19/3, *Syt1*^+/+^*_1xRNA(Syt1)_* 1.32 ± 0.1, *n* = 20/3, *p* = n.s., *Syt1*^+/+^_*2xRNA(Syt1*)_ 1.26 ± 0.09, *n* = 24/3, *p* = n.s., and *Syt1*^+/+^*_4xRNA(Syt1)_* 1.33 ± 0.10, *n* = 27/3, *p* = n.s.; *SYT1/VGLUT1* norm.: *Syt1*^+/+^ 1 ± 0.05, *n* = 19/3, *Syt1*^+/+^*_1xRNA(Syt1)_* 0.25 ± 0.04, *n* = 20/3, *p* < 0.001, *Syt1*^+/+^*_2xRNA(Syt1)_* 0.13 ± 0.02, *n* = 24/3, *p* < 0.0001, and *Syt1*^+/+^*_4xRNA(Syt1_*_)_ 0.096 ± 0.01, *n* = 27/3, *p* < 0.0001; Kruskal–Wallis test).

10.1523/JNEUROSCI.1945-21.2022.f7-1 Figure 7-1 Values corresponding to Figure 7-1 (***H–K***). Download Figure 7-1, DOCX file.

**Figure 8.**
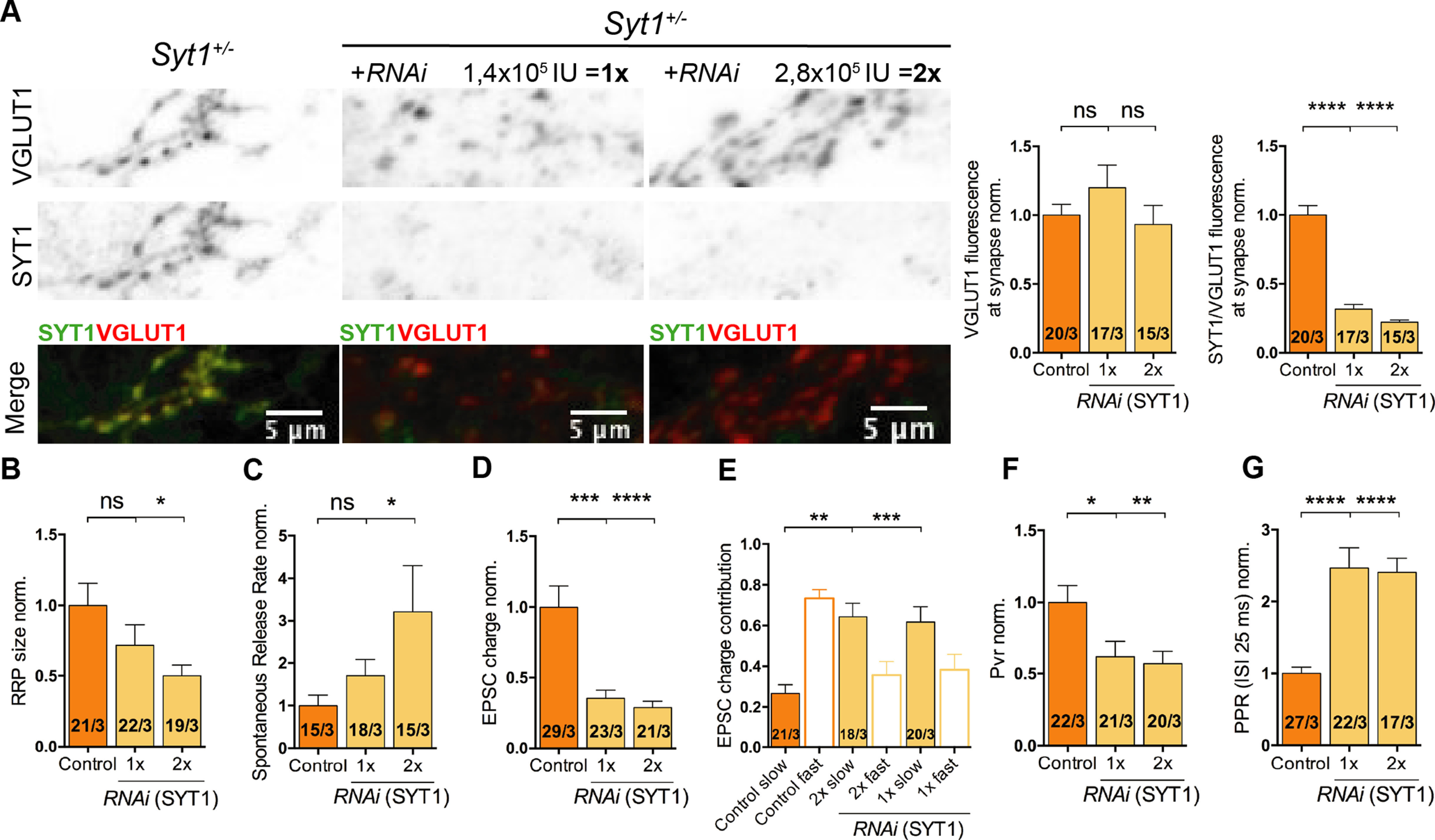
Impact of SYT1 RNAi viral titer on synaptic function in Synaptotagmin-1^+/−^ neurons. All immunocytochemistry and electrophysiological experiments were done from *Syt1*^+/−^ hippocampal glutamatergic autaptic neurons at DIV15–21. ***A***, Representative images of *Syt1*^+/−^ cultured hippocampal glutamatergic neurons infected with a lentiviral vector expressing either RNAi or scramble RNA as control. Scale bar, 5 µm. Left, Summary bar graph of VGLUT1 fluorescence at synaptic terminal. Right, Summary bar graph showing SYT1/VGLUT1 fluorescence at the synaptic terminal normalized to *Syt1*^+/+^. ***B***, Normalized bar graph of sucrose charge currents from autaptic cultures of Syt1^+/+^ neurons with *scRNA* (control) or RNA interference. ***C***, Summary bar graph of spontaneous release rate. ***D***, Normalized bar graphs of the effect of RNA interference on evoked EPSC charge. ***E***, Bar graphs of the relative contribution of the synchronic and asynchronic components to the EPSC total charge transfer after a single AP with different amount of SYT1 levels. ***F***, Vesicular release probability (Pvr) was calculated by the ratio of evoked EPSC charge and RRP size and normalized to *Syt1*^+/+^. ***G***, Normalized summary graph of the paired-pulse stimulation at 40 Hz. Statistical analysis was applied by Kruskal–Wallis test (*p ≤ 0.05, **p ≤ 0.01, ***p ≤ 0.001, ****p ≤ 0.0001). ns, Not significant. All data shown indicate mean ± SEM. Scale bars: 5 μm.

We found that the number of fusion-competent vesicles, as measured by sucrose application, was only significantly reduced when the SYT1 protein expression level was below 10% (Fig. 7*B*; RRP norm.: *Syt1*^+/+^ 1 ± 0.11, *n* = 51/4, *Syt1*^+/+^*_1xRNA(Syt1)_* 0.97 ± 0.13, *n* = 35/4, *p* = n.s., S*yt1*^+/+^*_2xRNA(Syt1)_* 0.83 ± 0.11, *n* = 37/4, *p* = n.s., and *Syt1*^+/+^*_4xRNA(Syt1)_* 0.61 ± 0.08, *n* = 39/4, *p* < 0.05; Kruskal–Wallis test). On the other hand, the spontaneous release rate was affected by the gradual reduction in SYT1 expression levels, showing progressive unclamping with reduction of SYT1 expression, reaching about a threefold increase when SYT1 levels were close to those of *Syt1*^−/−^ (Fig. 7*C*; Spontaneous rate norm.: *Syt1*^+/+^ 1 ± 0.11, *n* = 55/4, *Syt1*^+/+^*_1xRNA(Syt1)_* 1.54 ± 0.16, n = 40/4, *p* < 0.05, *Syt1*^+/+^*_2xRNA(Syt1)_* 1.67 ± 0.23, *n* = 35/4, *p* < 0.05, and *Syt1*^+/+^*_4xRNA(Syt1)_* 2.4 ± 0.47, *n* = 41/4, *p* < 0.0001; Kruskal–Wallis test). Processes involved in evoked release showed an even higher sensitivity to SYT1 expression levels (Fig. 7*D–G*). The EPSC charge as well as the fast synchronous component of release was significantly reduced with all levels of SYT1 knock down tested (Fig. 7*D*,*E*; EPSC charge norm.: *Syt1*^+/+^ 1 ± 0.11, *n* = 57/4, *Syt1*^+/+^*_1xRNA(Syt1)_* 0.58 ± 0.1, *n* = 36/4, *p* < 0.01, *Syt1*^+/+^*_2xRNA(Syt1)_* 0.36 ± 0.06, *n* = 39/4, *p* < 0.0001, and *Syt1*^+/+^*_4xRNA(Syt1)_* 0.32 ± 0.05, *n* = 43/4, *p* < 0.0001; Kruskal–Wallis test; EPSC components: *Syt1*^+/+^, slow 0.28 ± 0.02, fast 0.72 ± 0.02, *n* = 56/4, *Syt1*^+/+^*_1xRNA(Syt1_*_)_, slow 0.61 ± 0.06, fast 0.39 ± 0.06, *n* = 29/4, *p* < 0.0001, *Syt1*^+/+^*_2xRNA(Syt1)_*, slow 0.66 ± 0.04, fast 0.34 ± 0.04, *n* = 39/4, *p* < 0.0001, and *Syt1*^+/+^*_4xRNA(Syt1)_*, slow 0.65 ± 0.04, fast 0.35 ± 0.04, *n* = 40/4, *p* < 0.0001; one-way ANOVA test). Additionally, the release efficacy was significantly reduced in all knocked-down groups, demonstrated by a reduced Pvr and increased paired-pulse ratio compared with *Syt1*^+/+^ (Fig. 7*F*,*G*; Pvr norm.: *Syt1*^+/+^ 1 ± 0.08, *n* = 51/4, *Syt1*^+/+^*_1xRNA(Syt1_*_)_ 0.73 ± 0.12, *n* = 30/4, *p* < 0.01, *Syt1*^+/+^_*2xRNA(Syt1*)_ 0.52 ± 0.07, *n* = 31/4, *p* < 0.0001, and *Syt1*^+/+^*_4xRNA(Syt1)_* 0.5 ± 0.07, *n* = 39/4, *p* < 0.0001; PPR40Hz, norm.: *Syt1*^+/+^ 1 ± 0.05, *n* = 51/4, *Syt1*^+/+^*_1xRNA(Syt1)_* 1.84 ± 0.18, *n* = 34/4, *p* < 0.001, *Syt1*^+/+^*_2xRNA(Syt1)_* 2.16 ± 0.19, *n* = 33/4, *p* < 0.0001, and *Syt1*^+/+^*_4xRNA(Syt1)_* 2.67 ± 0.35, *n* = 39/4, *p* < 0.0001; Kruskal–Wallis test). To exclude that these results might be because of the number of viral particles used, we manipulated SYT1 endogenous protein expression in *Syt1*^+/−^ neurons (DIV15–21) using the same *Syt1 RNAi* construct (Fig. 8). We used half of the amount of viral infection units to reduce the expression to approximately the same levels as in the titration experiments in the *Syt1*^+/+^ neurons (Fig. 8*A*; *VGLUT1* norm.: *Syt1*^+/−^ 1 ± 0.08, *n* = 20/3, *Syt1*^+/−^ *_1xRNA(Syt1)_* 1.2 ± 0.16, *n* = 17/3, *p* = n.s., and *Syt1*^+/−^ *_2xRNA(Syt1)_* 0.93 ± 0.14, *n* = 15/3, *p* = n.s.; *SYT1/VGLUT1* norm.: *Syt1*^+/−^ 1 ± 0.07, *n* = 20/3, *Syt1*^+/−^*_1xRNA(Syt1)_* 0.32 ±0 .03, *n* = 17/3, *p* < 0.0001, and *Syt1*^+/−^ *_2xRNA(Syt1)_* 0.22 ± 0.02, *n* = 15/3, *p* < 0.0001; Kruskal–Wallis test). All electrophysiological parameters measured showed the same trend as our experiments performed on *Syt1*^+/+^ neurons (Fig. 8*B–G*; RRP norm.: *Syt1*^+/+^ 1 ± 0.16, *n* = 21/3, *Syt1*^+/+^*_1xRNA(Syt1)_* 0.72 ± 0.14, *n* = 22/3, *p* = n.s., and *Syt1*^+/+^*_2xRNA(Syt1)_* 0.5 ± 0.08, *n* = 19/3, *p* < 0.05; Spontaneous rate norm.: *Syt1*^+/+^ 1 ± 0.25, *n* = 15/3, *Syt1*^+/+^*_1xRNA(Syt1)_* 1.70 ± 0.39, *n* = 18/3, *p* = n.s., and *Syt1*^+/+^*_2xRNA(Syt1)_* 3.21 ± 1.09, *n* = 15/4, *p* < 0.05; EPSC charge norm.: *Syt1*^+/+^ 1 ± 0.15, *n* = 29/3, *Syt1*^+/+^*_1xRNA(Syt1)_* 0.35 ± 0.06, *n* = 23/3, *p* < 0.001, and *Syt1*^+/+^*_2xRNA(Syt1)_* 0.29 ± 0.05, *n* = 21/4, *p* < 0.0001; EPSC components: *Syt1*^+/+^, slow 0.27 ± 0.04, fast 0.73 ± 0.04, *n* = 21/3, *Syt1*^+/+^*_1xRNA(Syt1_*_)_, slow 0.62 ± 0.08, fast 0.38 ± 0.08, *n* = 18/3, *p* < 0.01, and *Syt1*^+/+^*_2xRNA(Syt1)_*, slow 0.64 ± 0.07, fast 0.36 ± 0.07, *n* = 20/3, *p* < 0.001; Pvr norm.: *Syt1*^+/+^ 1 ± 0.12, *n* = 22/3, *Syt1*^+/+^*_1xRNA(Syt1)_* 0.62 ± 0.11, *n* = 21/3, *p* < 0.05, and *Syt1*^+/+^*_2xRNA(Syt1)_* 0.57 ± 0.09, *n* = 20/3, *p* < 0.01; PPR40Hz, norm.: *Syt1*^+/+^ 1 ± 0.09, *n* = 27/3, *Syt1*^+/+^*_1xRNA(Syt1)_* 2.47 ± 0.28, *n* = 22/3, *p* < 0.0001, and *Syt1*^+/+^_*2xRNA(Syt1*)_ 2.41 ± 0.2, *n* = 17/3, *p* < 0.0001; Kruskal–Wallis test).

Our previous results suggest different relationships between synaptic functions and SYT1 expression levels. To understand these relationships in greater detail, we used our wide range of SYT1 protein level measurements at the presynaptic terminals of glutamatergic neurons (DIV15–21) to construct dose–response curves for the following different release properties: RRP size, spontaneous release rate, EPSC charge, and Pvr (Fig. 7*H–K*) and fitted our experimental data with a standard Hill equation (see above, Materials and Methods). To illustrate how SYT7 may potentially modulate the synaptic functions of SYT1 and to illustrate putative redundant function between SYT1 and SYT7, we included the normalized data from the SYT7 knock-down experiments performed on *Syt1*^−/−^ neurons from Figure 4 (DIV15–16). However, the SYT7 data point was excluded from the curve fitting of the SYT1 expression–function relation.

First, the function of SYT1 in synaptic vesicle priming was least sensitive to protein loss (Fig. 7*H*) because even an 85% decrease in SYT1 protein expression at the synapse still displayed normal priming function. The best-fit value Kd for this parameter of 0.02 demonstrates that only when SYT1 is almost absent from the synapse, RRP size decreases (Fig. 7*H*). The function of SYT1 as a regulator of spontaneous NT release (Fig. 7*I*) showed a higher sensitivity to protein levels, leading to increased spontaneous release activity when the expression levels were reduced by 50% or more. Evoked release was the most sensitive to the variations of SYT1 expression, as reflected both by assessing EPSC charge and Pvr (Fig. 7*J*,*K*). The similarity of the EPSC charge and Pvr functions is not surprising as both reflect the role of SYT1 as calcium sensor for evoked release. The Kd value (0.47) for the EPSC charge fit indicates that genetic modification of SYT1 expression associated with allelic loss or gene duplication may lead to a more pronounced impact on evoked neurotransmitter release compared with SYT1 functions in vesicle priming or suppression of spontaneous release. The difference in the sensitivity and shape of the dose–function relationships (Fig. 7*H*,*I*) may also indicate that SYT1 performs its distinct functions with different molecular stoichiometries.

## Discussion

In this study, we iterated the existence of three different synaptic functions performed by SYT1 using an autaptic primary neuronal culture model. Previous results concerning the ability of SYT1 to promote SV docking/priming (Geppert et al., 1994; Jorgensen et al., 1995; Reist et al., 1998; Nagy et al., 2006; Liu et al., 2009; Bacaj et al., 2013, 2015; Imig et al., 2014; Chang et al., 2018; Huson et al., 2020) and spontaneous release clamping (DiAntonio and Schwarz, 1994; Geppert et al., 1994; Littleton et al., 1994; Mackler et al., 2002; Yoshihara and Littleton, 2002; Chicka et al., 2008; Liu et al., 2009; Xu et al., 2009; Bacaj et al., 2013; Wierda and Sørensen, 2014) have been contradictory. Here, we demonstrated that SYT1 has a role in SV priming and clamping spontaneous release, which becomes essential over neuronal maturation. More mature SYT1-lacking hippocampal glutamatergic neurons showed a deficit in the RRP size and an increase in the spontaneous release rate, whereas early autaptic cultures did not (Table 1). Therefore, the time point and neuronal maturation stage at which experiments were performed could explain some discrepancies in previous work. What factor could compensate for SYT1 loss at early stages? We found that SYT7 has partially overlapping functions to SYT1 (Figs. 4, 7), largely consistent with previous results (Bacaj et al., 2013, 2015), and we conclude that at an early neuronal stage endogenous SYT7 protein could compensate for the loss of SYT1 in calcium-independent SV functions, such as priming and clamping of spontaneous release. When both proteins were missing at the presynaptic terminal, regardless of the culture stage, the pool of SVs was consistently reduced, and spontaneous release was significantly increased.

To dissect the specific interaction of the different mechanisms of NT release and to better understand the functions of SYT1 beyond that in synchronous release, we investigated the impact of systematically varying SYT1 expression levels on SV priming, clamping, and evoked release. SV priming was impaired only after a major drop in SYT1 concentration, suggesting that this process, although ultimately affected by the presence of SYT1, was relatively insensitive to changes in the number of SYT1 molecules. Varying SYT1 protein concentration also had a rather moderate impact on spontaneous release compared with the graded impact of varying SYT1 concentration on calcium-triggered release processes. Accordingly, heterozygotic *Syt1*^+/−^ excitatory hippocampal neurons with a 50% reduction in SYT1 protein expression in the presynaptic terminal showed no effect on RRP size compared with WT SYT1 protein levels, whereas calcium-dependent release efficacy was impaired by the 50% SYT1 protein reduction in *Syt1*^+/−^ neurons. Hence, when we applied a dose–response model to illustrate the sensitivity of the priming function to SYT1 expression, the curve described has a hyperbolic-like shape, where small amounts of SYT1 protein expression are sufficient to preserve maximum priming activity. Our overall analysis of the sensitivity of synaptic processes to SYT1 protein levels renders a rank where the least sensitive function is SV priming, followed by clamping of spontaneous and, finally, calcium-evoked release. Do the differences in sensitivity to SYT1 protein levels of these three synaptic processes indicate that SYT1 is involved in three consecutive and independent pathways? Because of the clear difference in sensitivity to expression levels for priming compare to evoked release, we think that SYT1 is affecting at least two pathways, in addition to its possible roles in endocytosis (Poskanzer et al., 2003; Yao et al., 2011; Liang et al., 2017). One hypothesis is that SYT1 undergoes different conformational stages to regulate different synaptic processes. Indeed, it has been suggested that SYT1 C2B domain-dependent oligomerization provides the molecular basis for SYT1 control of spontaneous and asynchronous release, and on influx of calcium SYT1 oligomers undergoes a conformational change from an SV clamping mode to a mode that allows the triggering of synchronous release (Bello et al., 2018; Tagliatti et al., 2020).

SYT1 protein level titration experiments not only revealed to us the distinct roles of SYT1 but may also could contribute to the understanding of the pathophysiological mechanisms underlying SYT1-associated neurodevelopmental disorders (Baker et al., 2015, 2018; Bradberry et al., 2020). In our study, we show that haploinsufficiency of SYT1 resulted in an aberrant spontaneous release phenotype and decreased release probability with additional consequences in short-term plasticity characteristics. If in patients with heterozygous SYT1 mutations, that have been described to produce a SYT1 loss of function (Bradberry et al., 2020), a reduced SYT1 protein level occurs, this could lead to an enhanced spontaneous release or a decreased release probability and, ultimately, contribute to network disfunction. It has been reported that at least one of the *de novo SYT1* mutations (*SYT1*_M303K_) is expressed at lower level than the endogenous wild-type protein and failed to localize at nerve terminals (Baker et al., 2018). Furthermore, two of the variants (*SYT1*_D304G_ and *SYT1*_D366E_) failed to efficiently relocalize to nerve terminals following stimulation (Baker et al., 2018). Although allelic expressivity may not explain the whole pathophysiology of the SYT1-associated neurodevelopmental disorder, it could exacerbate synaptic manifestations of individual SYT1 variants (Baker et al., 2018; Bradberry et al., 2020). In fact, a recent article revealed aberrant spontaneous NT release with some mutations from SNAP25-associated encephalopathies, indicating that when this form of release is affected it could result in developmental and epileptic encephalopathies (Alten et al., 2021). Conversely, our findings may indicate that the patient's pathophysiology that could derive from changes in the SYT1 expression levels are unlikely to be related to the priming of synaptic vesicles function at the presynaptic terminal.

Although the function of SYT1 as a calcium sensor for NT release has been extensively proven, the role for SYT7 is less clear. SYT7 has been suggested as the calcium sensor for asynchronous release (Maximov et al., 2008; Bacaj et al., 2013; Luo et al., 2015; Turecek and Regehr, 2018). SYT7 has also been proposed as a mediator of short-term facilitation of transmission during repetitive stimulation (Wen et al., 2010; Jackman et al., 2016; Chen et al., 2017b; Fujii et al., 2021), involving a mechanism of the concerted action of SYT7 with SYT1 on the fusion energy barrier (Schotten et al., 2015; Jackman and Regehr, 2017; Chen et al., 2017b; Huson et al., 2020). Supporting its role as a calcium sensor, SYT7 contributes to regulated exocytosis in chromaffin and pancreatic cells (Sugita et al., 2001; Shin et al., 2002; Schonn et al., 2008; Bendahmane et al., 2020). Additionally, it has been reported that SYT7 functions as a Ca^2+^ sensor for synaptic vesicle replenishment (Liu et al., 2014; Silva et al., 2021). In our study, we found no direct evidence that SYT7 is involved in regulating calcium-dependent release. However, we showed that suppressing SYT7 expression on *Syt1*^−/−^ neurons further decreased evoked neurotransmitter release, and the analysis of RRP size reveals that the reduction in evoked response is rather because of the priming action of SYT7 and not because of an effect on calcium-triggered release. It is quite possible that putative competition between SYT1 and SYT7 may contribute to the observed modulation of calcium-triggered NT release.

This study could be framed within a series of works that aim to understand how synapses respond to the relative changes in the expression of different presynaptic components and to study the consequences on neurotransmission (Arancillo et al., 2013; Zarebidaki et al., 2020). Although mammalian and invertebrate loss-of-function mutants of presynaptic proteins continue to provide deep insights into their role in neurotransmitter release (Geppert et al., 1994; Schulze et al., 1995; Deng et al., 2011), a more sophisticated model where there is a control of protein amount has been proven to be a powerful approach. For instance, examining the Munc13-1 concentration dependency of priming showed that the Rab3-interacting molecule boosts the priming function of Munc13-1 (Zarebidaki et al., 2020). Another example comes from the study of Syntaxin-1 (Stx1), where to titrate down protein expression levels of Stx1 revealed that priming and vesicle fusion are likely governed by highly related mechanisms (Arancillo et al., 2013). Here, therefore, we advocate for this experimental approach as a useful way to obtain a quantitative understanding of how the different elements of the presynaptic release machinery differently regulate the steps involved in the process of synaptic transmission.

Overall, to precisely understand the distinct functions of SYT1, variations in the expression and balance between SYT1 and SYT7 at SVs and the PM over neuronal stages should be taken into consideration. Also, our data demonstrated that both SYT1 and SYT7 are capable of SV priming and clamping. Likely the efficient execution of these functions relies on their intermolecular interactions with both SNAREs and phospholipids from the PM (Perin et al., 1990; Chapman et al., 1995; Li et al., 1995; Sugita et al., 2002; Zhou et al., 2017). Further study on how the interplay between these proteins affects the intrinsic speed of neurotransmitter release is needed. Moreover, we emphasize that when studying disorders associated with presynaptic protein mutations, all these parameters could be conditioning the quantitative aspects of the cellular phenotype and, in turn, the pathophysiology that derives from it.
