# Supplementary material for: Deconstructing Synaptotagmin-1's Distinct Roles in Synaptic Vesicle Priming and Neurotransmitter Release
Source: J Neurosci. 2022 Apr 6;42(14):2856–71. doi: 10.1523/JNEUROSCI.1945-21.2022 (PMC8985867; doi:10.1523/JNEUROSCI.1945-21.2022)
Supplement: Figure 7-1 — Values corresponding to Figure 7-1 (H–K). Download Figure 7-1, DOCX file. [file ns-JN-RM-1945-21-s01.docx]

Figure 7-1. Values corresponding to Figure 7H-K.

| H |  | SYT1 expression norm. | | RRP norm. |  | | | I | SYT1 expression norm. | | Spontaneous Release norm. | |
| --- | --- | --- | --- | --- | --- | --- | --- | --- | --- | --- | --- | --- |
| Syt1+/+ |  | 1,00 | 0,08 | 1 | | 0,072 | Syt1+/+ |  | 1,00 | 0,08 | 1,000 | 0,079 |
| Syt1+/- |  | 0,54 | 0,04 | 0,9808 | | 0,087 | Syt1+/- |  | 0,54 | 0,04 | 1,749 | 0,205 |
| Syt1-/- |  | 0,02 | 0,02 | 0,5469 | | 0,047 | Syt1-/- |  | 0,02 | 0,02 | 3,363 | 0,326 |
| Syt1+/+ | 1x *RNAi (SYT1)* | 0,25 | 0,04 | 0,9697 | | 0,127 | Syt1+/+ | 1x *RNAi (SYT1)* | 0,25 | 0,04 | 1,539 | 0,163 |
|  | 2x *RNAi (SYT1)* | 0,13 | 0,02 | 0,8282 | | 0,107 |  | 2x *RNAi (SYT1)* | 0,13 | 0,02 | 1,666 | 0,230 |
|  | 4x *RNAi (SYT1)* | 0,10 | 0,01 | 0,6067 | | 0,075 |  | 4x *RNAi (SYT1)* | 0,10 | 0,01 | 2,391 | 0,474 |
| Syt1+/+ | 1x SYT1 | 1,78 | 0,06 | 0,9109 | | 0,103 | Syt1+/+ | 1x SYT1 | 1,78 | 0,06 | 0,622 | 0,088 |
| Syt1-/- | *iRNA (SYT7)* | 0,01 | 0,03 | 0,08801 | | 0,016 | Syt1-/- | *iRNA (SYT7)* | 0,01 | 0,03 | 8,260 | 1,008 |
| J |  | SYT1 expression norm. | | EPSC charge norm. |  | | | K | SYT1 expression norm. | | Pvr norm. | |
| Syt1+/+ |  | 1,00 | 0,08 | 1 | | 0,076 | Syt1+/+ |  | 1,00 | 0,08 | 1,000 | 0,054 |
| Syt1+/- |  | 0,54 | 0,04 | 0,9808 | | 0,087 | Syt1+/- |  | 0,54 | 0,04 | 0,808 | 0,059 |
| Syt1-/- |  | 0,02 | 0,02 | 0,2176 | | 0,023 | Syt1-/- |  | 0,02 | 0,02 | 0,470 | 0,078 |
| Syt1+/+ | 1x *RNAi (SYT1)* | 0,25 | 0,04 | 0,5809 | | 0,099 | Syt1+/+ | 1x *RNAi (SYT1)* | 0,25 | 0,04 | 0,731 | 0,124 |
|  | 2x *RNAi (SYT1)* | 0,13 | 0,02 | 0,3553 | | 0,056 |  | 2x *RNAi (SYT1)* | 0,13 | 0,02 | 0,520 | 0,065 |
|  | 4x *RNAi (SYT1)* | 0,10 | 0,01 | 0,3208 | | 0,05 |  | 4x *RNAi (SYT1)* | 0,10 | 0,01 | 0,495 | 0,066 |
| Syt1+/+ | 1x SYT1 | 1,78 | 0,06 | 1,23 | | 0,129 | Syt1+/+ | 1x SYT1 | 1,78 | 0,06 | 1,196 | 0,144 |
| Syt1-/- | *iRNA (SYT7)* | 0,01 | 0,03 | 0,03991 | | 0,007 | Syt1-/- | *iRNA (SYT7)* | 0,01 | 0,03 | 0,399 | 0,102 |
